# Supplementary material for: Efficacy of a mixture of Ginkgo biloba, sesame, and turmeric on cognitive function in healthy adults: Study protocol for a randomized, double-blind, placebo-controlled trial
Source: PLoS One. 2023 Mar 15;18(3):e0280549. doi: 10.1371/journal.pone.0280549 (PMC10016651; doi:10.1371/journal.pone.0280549)
Supplement: S1 Checklist — (DOC) [file pone.0280549.s001.doc]

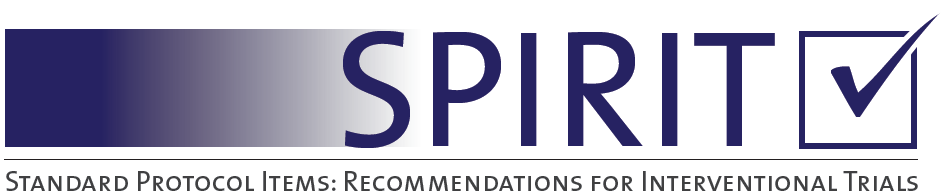


SPIRIT 2013 Checklist: Recommended items to address in a clinical trial protocol and related documents*

| Section/item | ItemNo | Description |
| --- | --- | --- |
| **Administrative information** | | |
| Title | 1 | Efficacy of a mixture of Ginkgo biloba, sesame, and turmeric on cognitive function in healthy adults: study protocol for a randomized, double-blind, placebo-controlled trial |
| Trial registration | 2a | UMIN000043494 |
|  | 2b | University Hospital Medical Information Network Clinical Trials Registry (UMIN-CTR) |
| Protocol version | 3 | August 7, 2021（6th edition） |
| Funding | 4 | This study is a joint research with Ohki Pharmaceutical Company and funding is provided by the same company. |
| Roles and responsibilities | 5a | Conceptualization: Taizen Nakase, Yasuyuki Taki.  Data curation: Taizen Nakase, Yasuko Tatewaki, Izumi Matsudaira, Kouki Kobayashi, Hikari Iki, Haruka Asaoka, Radiztia Ekayantri, Michiho Muranaka, Taeko Makino.  Formal analysis: Taizen Nakase, Yasuko Tatewaki, Yasuyuki Taki.  Funding acquisition: Hiroyuki Murata, Yasuyuki Taki.  Investigation: Taizen Nakase, Yasuko Tatewaki, Tatsushi Mutoh, Yasuyuki Taki.  Methodology: Taizen Nakase, Yasuyuki Taki.  Project administration: Taizen Nakase, Yasuyuki Taki. |
|  | 5b | Prof. Yasuyuki Taki  Department of Aging Research & Geriatric Medicine  Smart Aging Research Center, Tohoku Univertisy  〒980-8575  4-1 Seiryou Machi, Aoba ku, Sendai, Japan  TEL 022-717-8559　 FAX 022-717-8560  E-mail yasuyuki.taki.c7@tohoku.ac.jp |
|  | 5c | Role of study sponsor and funders: None. |
|  | 5d | This study does not have the coordinating centre, steering committee, endpoint adjudication committee, data management team, and other individuals or groups overseeing the trial. |
| Introduction |  |  |
| Background and rationale | 6a | Described in p3 line2 – line32. |
|  | 6b | Described in p4 line1 – line22. |
| Objectives | 7 | Described in p4 line22 – line26. |
| Trial design | 8 | Described in p4 line29 – p5 line3. |
| Methods: Participants, interventions, and outcomes | | |
| Study setting | 9 | Described in p5 line4 – line9.  Figure 1 and 2. |
| Eligibility criteria | 10 | Described in p5 line19 – line29. |
| Interventions | 11a | Described in p6 line11 – p7 line7.  Table 1. |
|  | 11b | Described in p7 line14 – line23. |
|  | 11c | Described in p8 line24 – line29. |
|  | 11d | Described in p8 line27 – line29. |
| Outcomes | 12 | Described in p5 line4 – line9. |
| Participant timeline | 13 | Described in p8 line1 – line23. |
| Sample size | 14 | Described in p6 line1 – line9. |
| Recruitment | 15 | Described in p5 line1 – line3. |
| **Methods: Assignment of interventions (for controlled trials)** | | |
| Allocation: |  |  |
| Sequence generation | 16a | Described in p7 line8 – line13. |
| Allocation concealment mechanism | 16b | Described in p7 line8 – line13. |
| Implementation | 16c | Described in p5 line3. |
| Blinding (masking) | 17a | Described in p7 line8 – line13. |
|  | 17b | Described in p7 line8 – line13. |
| **Methods: Data collection, management, and analysis** | | |
| Data collection methods | 18a | Described in p8 line5 – line23. |
|  | 18b | Described in p8 line5 – line23. |
| Data management | 19 | Described in p9 line2 – line4. |
| Statistical methods | 20a | Described in p9 line1 – line24. |
|  | 20b | N/A |
|  | 20c | Described in p7 line22 – line23. |
| **Methods: Monitoring** | | |
| Data monitoring | 21a | This study has only two assessments, that is, at baseline and at the endpoint. Therefore, it does not need a DMC. |
|  | 21b | Described in p7 line22 – line23. |
| Harms | 22 | Described in p7 line14 – line23. |
| Auditing | 23 | N/A |
| Ethics and dissemination | | |
| Research ethics approval | 24 | Described in p9 line25 – p10 line4. |
| Protocol amendments | 25 | Described in p10 line5 – line6. |
| Consent or assent | 26a | Described in p9 line32 – p10 line2. |
|  | 26b | This information is described in the informed consent form. |
| Confidentiality | 27 | Described in p10 line2 – line4. |
| Declaration of interests | 28 | N/A |
| Access to data | 29 | Described in p10 line2 – line4. |
| Ancillary and post-trial care | 30 | Described in p10 line6 – line11. |
| Dissemination policy | 31a | Described in p10 line13 – line15. |
|  | 31b | N/A |
|  | 31c | Described in p9 line25 – line29. |
| Appendices |  |  |
| Informed consent materials | 32 | Can be found in an attached file. |
| Biological specimens | 33 | N/A |

*It is strongly recommended that this checklist be read in conjunction with the SPIRIT 2013 Explanation & Elaboration for important clarification on the items. Amendments to the protocol should be tracked and dated. The SPIRIT checklist is copyrighted by the SPIRIT Group under the Creative Commons “[Attribution-NonCommercial-NoDerivs 3.0 Unported](http://www.creativecommons.org/licenses/by-nc-nd/3.0/)” license.
