# Supplementary material for: Efficacy of a mixture of Ginkgo biloba, sesame, and turmeric on cognitive function in healthy adults: Study protocol for a randomized, double-blind, placebo-controlled trial
Source: PLoS One. 2023 Mar 15;18(3):e0280549. doi: 10.1371/journal.pone.0280549 (PMC10016651; doi:10.1371/journal.pone.0280549)
Supplement: S1 File — (DOCX) [file pone.0280549.s002.docx]

第9版　2018年8月28日

| **TypeⅢ**  **Title**：  Association of the simultaneous administration of Ginkgo biloba extract and absorption promoting agent with cognitive function  （UMIN-CTR number：UMIN000043494）  Principal Investigator  Prof. Yasuyuki Taki  Department of Aging Research & Geriatric Medicine  Smart Aging Research Center, Tohoku Univertisy  〒980-8575  4-1 Seiryou Machi, Aoba ku, Sendai, Japan  TEL 022-717-8559　 FAX 022-717-8560  E-mail yasuyuki.taki.c7@tohoku.ac.jp  Study Office  Dr. Taizen Nakase  Department of Aging Research & Geriatric Medicine  Smart Aging Research Center, Tohoku Univertisy  〒 980-8575 4-1 Seiryou Machi, Aoba ku, Sendai, Japan  TEL 022-717-8559　 FAX 022-717-8560  E-mail taizen.nakase.a4@tohoku.ac.jp  August 7, 2021（6^th^ edition） |
| --- |

Contents

[1. Porpose 5](#_Toc122877463)

[2. Backgrounds and study rationale 5](#_Toc122877464)

[3. Backgrounds 5](#_Toc122877465)

[4. Scientific rationale 5](#_Toc122877466)

[5. Study design 6](#_Toc122877467)

[6. Study population 6](#_Toc122877468)

[7. Inclusion criteria 6](#_Toc122877469)

[8. Exclusion criteria 6](#_Toc122877470)

[9. Sample size and rationale 6](#_Toc122877471)

[10. Sample size 6](#_Toc122877472)

[11. Rationale 6](#_Toc122877473)

[12. Statistical analysis 7](#_Toc122877474)

[13. Statistical methods 7](#_Toc122877475)

[14. Methos and period of the study 7](#_Toc122877476)

[15. Study design 7](#_Toc122877477)

[16. Methods 8](#_Toc122877478)

[17. Study period 9](#_Toc122877479)

[18. Items and methods of evaluation 9](#_Toc122877480)

[19. Primary endpoint 9](#_Toc122877481)

[20. Secondary endpoint 9](#_Toc122877482)

[21. Data management, self-assessment 10](#_Toc122877483)

[22. Creation of Case Report Form (CRF) 10](#_Toc122877484)

[23. Self-assessment of CRF 10](#_Toc122877485)

[24. Trasfar and storage of CRF 10](#_Toc122877486)

[25. Procedure of CRF correction 10](#_Toc122877487)

[26. Competing interests of fundings 10](#_Toc122877488)

[27. Description of contract company 11](#_Toc122877489)

[28. Management of personal information 11](#_Toc122877490)

[29. Application purpose of personal information 11](#_Toc122877491)

[30. Utilization method （Anonymization methods） 11](#_Toc122877492)

[31. The safety management responsibility system （Security control action） 12](#_Toc122877493)

[32. Procedure of informed consent 13](#_Toc122877494)

[33. Informed consent to participants 13](#_Toc122877495)

[34. Agreement 13](#_Toc122877496)

[35. Procedures for receiving informed consent from a substitute, etc. 14](#_Toc122877497)

[36. Procedures for obtaining an informed ascent 14](#_Toc122877498)

[37. Procedures of disclosure 14](#_Toc122877499)

[38. Preparation and storage of records related to the transfer of samples and information 14](#_Toc122877500)

[39. Burdens, foreseeable risks (including possible adverse events) and benefits, comprehensive assessments of these subjects, and counterplan of minimizing burdens and risks 14](#_Toc122877501)

[40. Responding to consultations etc. from study participants etc. 15](#_Toc122877502)

[41. The fact and the contents, if there is an economic burden or reward for the study participants 16](#_Toc122877503)

[42. Assessment of adverse events 16](#_Toc122877504)

[43. Obtaining information 16](#_Toc122877505)

[44. Description of adverse events 17](#_Toc122877506)

[45. Response to the serious adverse event / incidents (including adverse events to be reported to head of the research institute) 18](#_Toc122877507)

[46. Response to the adverse event / incident 18](#_Toc122877508)

[47. Report to head of the research institute and principal investigator (research director) 18](#_Toc122877509)

[48. Report to joint research institution 19](#_Toc122877510)

[49. In case of invasive study, describe the existence and contents of compensation for health damage caused by the research 19](#_Toc122877511)

[50. When there is a possibility that important knowledge such as health of a research participant and genetic characteristics that can be passed down to descendants may be obtained with the implementation of the research, including incidental findings 19](#_Toc122877512)

[51. Potential use of samples and information for future research that is not identified at the time of consent / A possibility of providing it to other research institutions 20](#_Toc122877513)

[52. How to publish information about the study 20](#_Toc122877514)

[53. Registration of research outlines and results 20](#_Toc122877515)

[54. Publication of research results 20](#_Toc122877516)

[55. Method of storage and disposal of samples, information, etc. 20](#_Toc122877517)

[56. Strage 20](#_Toc122877518)

[57. Disporsal 21](#_Toc122877519)

[58. What and how to report to the head of a research institute 21](#_Toc122877520)

[59. Modification of the study protocol 21](#_Toc122877521)

[60. Research implementation system 22](#_Toc122877522)

[61. Name of research institution, name of research director 22](#_Toc122877523)

[62. Name and role of research allotment person, etc. 22](#_Toc122877524)

[63. Research Secretariat, Statistical Analysis 22](#_Toc122877525)

[64. Joint Research Institutes 22](#_Toc122877526)

[65. Contact point for inquiries about research 22](#_Toc122877527)

[66. References 22](#_Toc122877528)

Abstract

- 1. **Schema**

Eligibility

Healthy adults

Aged between 20 and 64 years-old

Both males and females

MoCA-J ≧26

Standard blood test and urine test

Body measurements, grasping power of both the hands, two-step test, and functional reach test

Wechsler Memory Scale-Revised, digit span, tapping span, Stroop Color Test, and Trail Making Test

T1-weighted MRI and rs-fMRI

24w

D 0

Placebo

GBE

GBE/MST

- 1. **Purpose**

Ginkgo biloba extract (GBE) is available in the form of health supplements in Japan, with claims of ameliorating memory and attention and mitigating the symptoms of Alzheimer’s disease. However, its efficacy in the cognitive function of healthy adults remains controversial. It was reported that concentrations of terpene lactones, active components of GBE that are present in very low concentrations in the brain, were significantly increased following administration of a mixture of GBE, sesame extract, and turmeric oil (GBE/MST) in mice. This study aims to investigate the effectiveness of GBE/MST on the cognitive function of healthy adults. For biological assessment, resting state functional magnetic resonance imaging (rs-fMRI) will be performed simultaneously with the neuropsychological tests.

- 1. **Subjects**

１）Inclusion criteria

- Male and female participants aged between 20 and 64 years
- Normal cognitive function at baseline, confirmed by the Japanese version of the Montreal Cognitive Assessment battery (MoCA-J) (score ≧26)

２）Exclusion criteria

1. History of severe neurological diseases
2. History of critical bleeding incidents
3. Use of antithrombotic medications
4. Severe depression
5. Diabetes mellitus
6. Severe visual or auditory disability
7. Metal implantation such as cardiac pacemaker, coronary stent, and artificial joints
8. Claustrophobia
9. Use of any supplements
10. Creatinine clearance below 15 mL/min
11. Gastrointestinal diseases or metabolic diseases
12. Diagnosed psychiatric disorders
13. Participants who cannot provide informed consent
14. Allergy to the study materials
15. Individuals not suitable for participation by the decision of the principal investigator
    1. **Sample size and period**
16. Sample size：159（control 53、GBE only 53、GBE/MST 53）、randomized, double-blind
17. Study period：April 2021（post ethical approval）～December 2022

（registration：～February 2022、follow up：～August 2022）

**0.5. Office**

1. Eligibility criteria and clinical decisions：

Department of Aging Research & Geriatric Medicine

Smart Aging Research Center, Tohoku Univertisy

〒 980-8575 4-1 Seiryou Machi, Aoba ku, Sendai, Japan

TEL 022-717-8559 FAX 022-717-856

1. Registration procedure, inspection checklist, etc:

Department of Aging Research & Geriatric Medicine

Smart Aging Research Center, Tohoku Univertisy

〒 980-8575 4-1 Seiryou Machi, Aoba ku, Sendai, Japan

TEL 022-717-8559 FAX 022-717-8560

# Porpose

This study aims to investigate the effectiveness of a mixture of Ginkgo biloba extract (GBE), sesame extract, and turmeric oil (GBE/MST) on the cognitive function of healthy adults.

# Backgrounds and study rationale

# Backgrounds

GBE is available in the form of health supplements in Japan and the USA, with claims of ameliorating memory and attention and mitigating the symptoms of Alzheimer’s disease. However, its efficacy in the cognitive function of healthy adults remains controversial. No effect was reported on memory enhancement and on the intelligence test scores after 6 days of GBE administration in healthy adults aged 20 years [1]. Reportedly, healthy elderly individuals showed no improvement in memory but showed improvements in executive functions after 6 weeks of GBE administration [2]. There was no difference in the prevalence rate of cognitive decline between the GBE and placebo groups for 42 months among healthy adults aged over 85 years. However, among participants who maintained good adherence throughout the study period, GBE was associated with a significantly reduced rate of cognitive decline when compared with placebo [3].

GBE has two active components, flavonoids and terpene lactones. Flavonoids have been reported to accumulate in the blood serum [4]. Since major effects of flavonoids include reduction in the inflammatory response, oxidization, and platelet aggregation in vivo, these effects are expected to play important roles in inhibiting damage to the vascular endothelial cells and improving the peripheral blood flow [5]. Terpene lactones have been reported to infiltrate the central nervous system (CNS) but are quickly metabolized, resulting in very low concentrations in the CNS [4]. Since terpene lactones were reported to reduce caspase-3 activation and amyloid-β aggregation and protect the mitochondrial membrane, they can be expected to protect the neurons from apoptosis and amyloid-β toxicity [6]. However, since the concentration of terpene lactones in the CNS might be very low in vivo [4, 7], the effect of GBE on cognitive function may be exclusively explained by improvement in the cerebral blood flow. Administration of a mixture of GBE, sesame seed extract, and turmeric oil (GBE/MST) was reported to increase the concentration of terpene lactones in the mouse brain [7]. The aforementioned study reported that the concentration of bilobalide, a terpene lactone, in the brain was not significantly different between the GBE alone and GBE/MST groups (mean ± standard error of the mean: 1.30±0.31 and 0.75±0.24 μg/mg, respectively). Ginkgolide A, another terpene lactone, was detected in the GBE/MST group, but it was not detected in the GBE alone group. The GBE/MST supplement is currently available in the Japanese market, although its scientific effects have not been validated. Since low bioavailability of GBE in the brain may be a possible cause of its faint effect on the cognitive function, it might be of great interest to investigate whether the efficacy of GBE is improved by administration in the form of GBE/MST in healthy adults.

# Scientific rationale

According to a meta-analysis involving nine clinical studies [8], a significant improvement was observed in the Syndrome-Kurztest scores following GBE administration in patients with Alzheimer’s disease. There was no significant difference in Alzheimer’s Disease Assessment Scale-Cognitive Subscale scores between patients consuming GBE and those consuming placebo. However, this difference was greater among patients consuming GBE in a dose of 240 mg/day compared to those on a dose of 120 mg/day. The difference was also greater in the observation period of 22-24 weeks compared to a period of 12 weeks. There was no difference in the adverse incident rate between GBE dose of 120 mg/day and 240 mg/day. Moreover, there was no difference in the dropout rate due to adverse effects between GBE doses of 120-160 mg/day or 240 mg/day and placebo [8, 9].

# Study design

# Study population

A population is healthy adults.

# Inclusion criteria

1. Male and female participants aged between 20 and 64 years at baseline
2. With informed consent
3. Normal cognitive function at baseline, confirmed by the Japanese version of the Montreal Cognitive Assessment battery (MoCA-J) (score≧26)

# Exclusion criteria

1. History of severe neurological diseases
2. History of critical bleeding incidents
3. Use of antithrombotic medications
4. Severe depression
5. Diabetes mellitus
6. Severe visual or auditory disability
7. Metal implantation such as cardiac pacemaker, coronary stent, and artificial joints
8. Claustrophobia
9. Use of any supplements
10. Creatinine clearance below 15 mL/min
11. Gastrointestinal diseases or metabolic diseases
12. Diagnosed psychiatric disorders
13. Participants who cannot provide informed consent
14. Allergy to the study materials
15. Individuals not suitable for participation by the decision of the principal investigator

# Sample size and rationale

# Sample size

Estimated sample number: 159（Control 53, GBE 53, GBE/MST 53）

# Rationale

The primary endpoint of this study is to determine the impact of GBE/MST on cognitive function in healthy adults, which will be assessed by the Wechsler Memory Scale-Revised (WMS-R) after 24 weeks of GBE/MST administration. The WMS-R is designed to indicate a standard deviation of 15 if its normal average score is 100. In the present study, when the estimated alteration rate of the standard deviation was 1%, the difference in the average score was 6. Cohen’s calculation formula with an effect size of 0.4 at a significance level (α) of 0.05 and a detection power (1-β) of 80% yielded a sample size of 44 participants. Considering a dropout rate of 20%, 159 participants will be required (53 participants in each group).

# Statistical analysis

# Statistical methods

(1) Analysis of cognitive function

The average of total WMS-R score at the endpoint will be compared among the GBE/MST, GBE, and placebo groups using one-way analysis of variance (ANOVA) and Tukey's test. The difference between the total score at baseline and at the endpoint will be compared among the three groups using multivariate ANOVA (MANOVA).

(2) Analysis of brain morphology

Total cerebral cortical volume will be calculated from the T1-weighted structural images using the SPM12 software. The average alteration in total volume from baseline to the endpoint will be compared among the three groups. Using the CONN toolbox (https://web.conn-toolbox.org/) implemented in MATLAB, functional connectivity (FC) between two regions of interest (ROIs) within the DMN will be analyzed by means of ROI-to-ROI analysis.

# Methos and period of the study

# Study design

1. Study design
   1. **Control**

Placebo

- 1. **Randomization**

Double-blind

- 1. **Comparison**

Randamized placebo-controlled

1. **Rationale of the study design**

The primary aim of this study is to determine the efficacy of GBE/MST in the impact of cognitive function when compared with that of GBE alone and placebo in healthy adults using neuropsychological assessments. The study also aims to evaluate the alterations in the default mode network (DMN) after 24 weeks of GBE/MST administration and compare them with the baseline data.

# Methods

1. Intervention

〇Active

・GBE/MST capsule (commercially known as “Brain assist ®︎”)

Ginkgo biloba extract, sesame oil, Piper longum extract, reduced sugar syrup, refined fish oil containing docosahexaenoic acid with gelatin, spice extract, glycerin, glycerin fatty acid ester, beeswax, caramel color, vegetable lecithin from soybean

4 kcal, proteins: 0.2g, lipids: 0.3g, carbohydrates: 0.2g, salt: 0.0008g

・GBE capsule (commercially known as “Icho-ba Ekisu”)

Ginkgo biloba extract, reduced sugar syrup, gelatin, glycerin fatty acid ester, caramel color, tocotrienol lecithin from soybean

5 kcal, proteins: 0.2g, lipids: 0.4g, carbohydrates: 0.2g, salt: 0.0009g

Both have a dosage of two capsules per day. Both the products will be provided by Ohki Pharmaceutical Company and will have similar shapes, colors, and tastes. The package containing the products will not have any information to avoid participant bias.

〇Placebo

Placebo will be made in matched for size, color and taste to the GBE/MST capsule and GBE capsule.

Reduced sugar syrup, refined fish oil containing docosahexaenoic acid with gelatin, spice extract, glycerin, glycerin fatty acid ester, beeswax, caramel color, vegetable lecithin from soybean

5 kcal, proteins: 0.2g, lipids: 0.5g, carbohydrates: 0.1g, salt: 0.0009g

1. **Dosage and administration**

Participants in the GBE/MST group will consume two capsules of the GBE/MST supplement. Participants in the GBE group will consume two capsules of GBE supplement. Participants in the placebo group will consume two capsules of placebo.

1. **Adverse effects and cessation criteria**

The adverse effects will be screened throughout the study period. Headache, dizziness, tinnitus, diarrhea, nausea, gastrointestinal symptoms, high blood pressure, excessive thirst, dyspnea, chest pain, bradycardia, and upper respiratory infection have been reported as adverse effects of GBE [5]. Interestingly, participants consuming GBE showed a lower incidence of adverse events when compared with those consuming a placebo [10]. The study will be terminated if any grade 3 adverse event is reported. If a participant shows any adverse events and requests a refusal, the study will be terminated.

1. **Schedule**

At registration, written informed consent will be obtained from the participants. Subsequently, cognitive function will be evaluated using the MoCA-J. Participants with a score of ≧26 will be eligible for inclusion.

Standard blood and urine tests will be performed to check for health status. Body measurements, grasping power of both the hands, two-step test, and functional reach test will be performed at baseline and at the endpoint (24 weeks).

Cognitive tests will be completed by the participants at baseline and at the endpoint. The WMS-R and its lower domains will be used to measure total memory function and assess individual function (digit span indicates verbal memory and the tapping span indicates visual memory). Stroop Color and Word Test and Trail Making Test will be employed to assess executive functions such as restraint-related functions and sustained attention, respectively.

Magnetic resonance imaging (MRI: Achieva Intera 3T, Philips Healthcare, Quasar Dual, The Netherlands) will be performed at baseline and at the endpoint. T1 weighted magnetization-prepared rapid acquisition gradient echo (MPRAGE) structural images (matrix: 240 × 240, repetition time: 8.70 ms, echo time: 3.1 ms, flip angle: 8°, field of view: 256 × 256 × 180 mm, 162 slices, voxel size: 0.7 × 0.7 × 0.7 mm, and scan duration: 5 min 15 s) will be acquired to collect 3D structural datasets. Resting state functional MRI (rs-fMRI: T2*-weighted gradient-echo planar imaging, matrix: 64× 64, repetition time: 3000 ms, echo time: 30 ms, flip angle: 90°, field of view: 220 × 220 ×218 mm, 34 slices, voxel size: 3.44 × 3.44 × 3.40 mm and 197 volumes with 5 min superimposition) with closed eyes will be performed to analyze the DMN.

# Study period

June 2021（post ethical approval）~ December 2022

# Items and methods of evaluation

# Primary endpoint

　To determine the efficacy of GBE/MST in the impact of cognitive function, data of GBE/MST will compare with that of GBE alone and placebo in healthy adults by the Wechsler Memory Scale-Revised (WMS-R) after 24 weeks.

# Secondary endpoint

1．Cognitive assessment

　a) MoCA-J …Screening of cognitive function

b) Trail Making Test-A, B　　…Screening of attention

c) Stroop test　　…Screening of frontal function

d) Tapping Span test　　…Screening of visual memory

　e) Digit Span test　　…Screening of verval memory

2．Brain MRI examination

The study also aims to evaluate the alterations in the default mode network (DMN) after 24 weeks of GBE/MST administration and compare them with the baseline data.

3．Physical assessment

　a)　 hight, body weight

　b)　 hand grip power、2 Step Test、Functional Reach Test

# Data management, self-assessment

# Creation of Case Report Form (CRF)

Co-investigators etc. will complete a CRF. Creation of CRF will be performed immediately after each examination. If there will be any mistake in a CRF, an investigator must report it to the study office.

# Self-assessment of CRF

1. Co-investigators etc. must validate details of each category in a CRF.
2. If there is any contradiction in a CRF, a document which explains the reason of contradiction must be created.
3. A principal investigator and co-investigators etc. must sign after validation and confirmation of a completed CRF.

# Trasfar and storage of CRF

A principal investigator must transfar the completed CRF to the study office following a fixed procedure, and store a copy of the completed CRF.

**（CRF submission office）**

Department of Aging Research & Geriatric Medicine

Smart Aging Research Center, Tohoku Univertisy

〒 980-8575 4-1 Seiryou Machi, Aoba ku, Sendai, Japan

TEL 022-717-8559 FAX 022-717-8560

# Procedure of CRF correction

If a CRF needs to be corrected, a principal investigator must submit a document of any change or correction in a CRF to the study office, and store a copy of the document.

# Competing interests of fundings

This study is a joint research with Ohki Pharmaceutical Company and Tohoku University Knowledgecast※, and funding is provided by Ohki Parmaceutical company. GBE/MST supplement, GBE supplement, and placebo will also be provided by this pharmaceutical company.

COI of this study must be described in the informed consent form, following the Declaration of Helsinki (World Medical Association) and the Ethical Guidelines for Medical and Biological Research Involving Human Subjects (Ministry of Health, Labour and Welfare, Japan). COI management will follow the guideline in each institution. COI of researchers in Tohoku University will be controlled by COI management committee, Tohoku University.

The funders have no role in the study design, data collection and analysis, decision to publish, or preparation of the manuscript.

※ This is an affiliated company of Tohoku University. Description of this company is to promote social utilization of research outcomes, consultation, and human resources development.

# Description of contract company

Product management and distribution is performed by Ohki Pharmaceutical Company.

# Management of personal information

# Application purpose of personal information

For obtaining appropriate results, the personal information will be stored both during study period and follow up period.

# Utilization method （Anonymization methods）

**（１）Criteria of personal information**

| subject | | definition | example | existence |
| --- | --- | --- | --- | --- |
| Personal information | | 1. Objects which can identify a specific individual by itself | Name, Picture of face, etc. | ■yes  □no |
|  |  | 1. Objects which can identify a specific individual by matching with other information | Data using a correspondence table |  |
|  |  | 1. Those containing a personal identification code | Genom data, etc. | □yes  （specify：　　　）  ■no |
|  | Special care-required personal information | Those containing a race, creed, social status, medical history, criminal record, fact of having suffered damage by a crime, or other descriptions prescribed by cabinet order as those of which the handling requires special care so as not to cause unfair discrimination, prejudice or other disadvantages to the person. | Medical record, Medical receipt, Medical examination data, Partial genom data* | ■yes  （specify：laboratory data）  □no |

* Genom data with medical information, such as single nucleotide polymorphism, monogenic disease, and sensitivity of medication, may be applicable in personal information with consideration.

**（２）Anonymization**

■yes（to（3））

□no（reason：　　　　　　　）

□other（specify：　example：non-identifiable processed information prescribed in the Act on the Protection of Personal Information Held by Incorporated Administrative Agencies）

**（３）Type and method of anlnymization**

■1）Anonymized（with the correspondence table for identifiable of a certain person stored in Tohoku University）

Method：The correspondence table in which personal information such as name and identification number will be deleted and alternative code or number will be addressed is produced in an institution of Tohoku University. A personal information manager appropriately stores it so as not to leak to the outside.

□2）Anonymized（those of deidentified, and the correspondence table is stored in another facility）

　Method：The correspondence table in which personal information such as name and identification number will be deleted and alternative code or number will be addressed is not stored in an institution of Tohoku University (The correspondence table is stored in another facility and is not stored in Tohoku University). If the correspondence table is stored in another institution of Tohoku University, this case is not applicable.

□3）Anonymized（those of deidentified and without the correspondence table）

Method：The correspondence table in which personal information such as name and identification number will be deleted and alternative code or number will be addressed is not produced.（The codrrespondence table is not produced in any facilities in this study）

□4）Other　（specify：　　　　）

# The safety management responsibility system （Security control action）

Personal information manager in Tohoku University

　・Name：Taizen Nakase

　・Affiliation：Department of Aging Research & Geriatric Medicine, Smart Aging Research Center

　・Qualification：Medical doctor

Security method：Storage with paper or electronic medium. Anonymized correspondence table and raw data are separately and appropriately stored.

Including 4 issues described below:

　・Physical safety control (A data management PC is securely stored in a rocked area of a laboratory in the department. Prohibit transfering strage medium. Act prevention of robbery and leakage.）

　・Technical safety control (Restriction of the access to a data management PC. Software security against injustice access from outside of the facility）

　・Organization safety control (Restrict the access of personal information and the authority to the principal investigator and co-investigators）

　・Human safety control (Periodic education)

# Procedure of informed consent

# Informed consent to participants

Investigators must present and explain the informed consent document which was apploved by the institutional organization.

**（Matters to be included）**

① Name of the study, and description about approvement of the study by the institute director

② Name of the institutional organization and principal investigator

③ Explanation of the purpose and significance of the study

④ Procedures and expected duration of the study

⑤ Explanation of selected reason as a study participant

⑥ Description of any burdens, foreseeable risks, and benefits for a study participant

⑦ Disclosure of the right to withdraw the agreement with participation to the study

⑧ Statement that participation is voluntary, refusal to participate will involve no penalty or loss of benefits, and a participant can discontinue participation at any time without penalty or loss of benefits

⑨ Procedures of the disclosure of study information

⑩ Explanation of obtaining and viewing study information those will not affect the personal information of other participants and the study originality

⑪ Procedures of handling personal informations（including the method when anonymizing）

⑫ Methods of storing and discarding study samples and information

⑬ Conflict of interest situation (source of funding, foreseeing conflicts of interest, and association with relevant organizations such as researchers）

⑭ Explanation of responding to consultations etc. from study participants and relatives etc.

⑮ Contents when there is any economic burden or reward for the study participants etc.

⑯ Explanation of handling any personal results (including incidental findings) in which this study may potentially obtain critical knowledge, such as personal health status and heritable genetic features

⑰ Explamation of any supports and contents, if applicable, against health problems caused by the study, in case of the study contains any invasive procedures

⑱ Explanation of any possibilities in those the samples and information obtained from study participants may be used in an unspecified future study or may be provided to other research institutes

# Agreement

Reserchers must explain the research, give them time to think carefully, confirm that the study participants have a good understanding of the content of the reserach, and then ask them to participate in the study. If the study participant agrees to participate in the examination, the consent form will be signed by oneself.

The original documents are stored by the study director of the research institute and a copy is passed to the study participant.

# Procedures for receiving informed consent from a substitute, etc.

A substitute is not allowed. The informed consent must be obtained from the principal.

# Procedures for obtaining an informed ascent

　N/A

# Procedures of disclosure

N/A

# Preparation and storage of records related to the transfer of samples and information

N/A

# Burdens, foreseeable risks (including possible adverse events) and benefits, comprehensive assessments of these subjects, and counterplan of minimizing burdens and risks

1. **Predicted benefits**

Study participants may get a partial health information, as the MRI findings will be checked by radiology specialists and the results will be provided.

1. **Foreseeable risks**

There are foreseeable risks and disadvantage regarding performed examinations, a temporal restriction of participating in the examinations, and an expected economical burden by using public transportation.

Risks and adverse events regarding blood sampling

Procedure of blood sampling will be the same as that performed in a health chek-up. Foreseeable events will be described below:

1. Bleeding from the site of puncture will be seen, and blueing may be expected. Counterplan is to perform complete astriction for 5min.

2. Tape eruption of skin may be occured because of a patch used on the site of puncture. Counterplan is to be asked if a participant had a risk of tape eruption. In that case, vinil tape will be used, instead of normal tape.

3. There is a possibility of allergic response by using alcohol rubbing cotton. Counterplan is to be asked about the existence of alcohol allergy. In that case, chlorhexidine swab will be used instead of alcohol swab.

4. There is a possibility of vasovagal reflex by pain or stress causing bad feeling and consciousness loss. Counterplan is to be asked as if a participant had a risk of vasovagal reflex before blood sampling. A participant may have a rest after blood sampling.

5. There is a possibility of the nerve injury along with blood sampling. The symptom of nerve injury is finger numbness and pain caused by injured peripheral nerves by needle puncture. The frequency is reported about 1/6000. Because it is anatomically difficult to completely prevent it, the blood sampling will be performed at an outpatient site following a recommended procedure as the ideal prevention. In case of the incident, a participant may consult a clinic using own medical insurance.

Risks and adverse events regarding MRI

1. The MRI apparatus may cause injury by iron materials existing in or on the body. Counterplans are described below:

・Ask several times about operation history, internal metals, and cardiac pacemaker, by means of document and interview.

・Confirm no metals on the body befor the examination.

・Inform that the examination can stop anytime during the procedure if a participant will feel any abnormalities.

2. Since the MRI apparatus has strong magnetic field, metallic materials can be pulled and may physically injure a participant. Counterplans are described below:

・Metallic material without anchoring must not be stored in the MRI room.

・The exclusinve stuffs of MRI must control the procedures in the MRI room which has protective methods against expecting risks.

3. There is a possibility of finding any critical diseases, such as brain tumore, brain absess, brain hemorrhage, brain infarction, brain artery aneurysm, arteriovenous malformation, etc., by analysis of MRI. If any of such abnormality will be found, an investigator will inform the participant about to ask a doctor.

4. During MRI procedures, undesired noise, magnetic field, change of body temperature, and unconfortable position in narrow space may cause discomfort. As counterplan of discomfort, a participant can use a buzzer for alart any bad feeling. If a buzzer sounds, an operator must stop the procedure, and a doctor must check a participant’s condition. In case of difficultiness, the MRI procedure can be cancelled.

Risks and adverse events regarding GBE and GBE/MST

　GBE, sesame extract, and turmeric oil have been used as suppliments, and their safety has been established. No critical adverse event was reported. Digestive symptoms and thirsty have been reported as side effects. These symptoms will diminish after 3-4 days of cessation. If such side effects are strong, participation of this study can immediately be stopped.

# Responding to consultations etc. from study participants etc.

　Contact information for general research

Taizen Nakase

Department of Aging Research & Geriatric Medicine

Smart Aging Research Center, Tohoku Univertisy

〒 980-8575 4-1 Seiryou Machi, Aoba ku, Sendai, Japan

TEL 022-717-8559 FAX 022-717-8560

Contact information for inquiries about privacy policy

Taizen Nakase

Department of Aging Research & Geriatric Medicine

Smart Aging Research Center, Tohoku Univertisy

〒 980-8575 4-1 Seiryou Machi, Aoba ku, Sendai, Japan

TEL 022-717-8559 FAX 022-717-8560

# The fact and the contents, if there is an economic burden or reward for the study participants

There is a possible disadvantage regarding participation of the examinations, i.e. a temporal restriction of participating in the examinations and an economical burden by using public transportation.

- Since GBE/MST, GBE, and placebo are provided from Ohki Pharmaceutical Company without compensation, study participants have no cost allocation.
- Expense of MRI and neuropsychiatric examinations is beared by the company.

Reward of 10,000 yen will be paid to the participants who completed all examinations (at registration and at 24 weeks). If a participant may stop the study, partial reward may be paid.

# Assessment of adverse events

# Obtaining information

1. Investigators etc. must deal with any critical adverse events or incidences, and report them to a principal investigator.
2. A principal investigator must confirm items below to investigators.

**Confirmation items to investigators etc.**

| - 1. Name of adverse event / incidence   2. Classification of severity^1)^   3. Seriousness^2)^, reason   4. Predictivity (unknown, known)^3)^   5. Relation to intervention (medicine / medical apparatus)   6. Details of the effect / incidence (date, history, outcome, etc.)   7. Personal information of the participant (initial, age, sex) |
| --- |

**^1)^ Classification of severity**

For evaluation of adverse event / incident, Japanese version of NCI-CommonTerminology Criteria for Adverse Events v4.0 (CTCAE v4.0-JCOG) is used.

About the CTCAE v4.0-JCOG items of which grade is defined as an institutional standard, “JCOG reference range” will be adopted instead of each institutional standard. Precise information about “JCOG reference range” is available in the website (http://www.jcog.jp/doctor/tool/kijun.html）.

If there is no applicable grade in the NCI CTCAE grade, decision will be performed following the **Severity classification standard for adverse events**.

| **NCI CTCAE Grade** | **Severity classification standard for adverse events** |
| --- | --- |
| **Grade1** | No or mild symptom. Only clinical or laboratorical abnormality.  No needs to treat. |
| **Grade2** | Minimal/partial/non-invasive treatment.  Restriction of instrumental ADL*, except for age-appropriate personal activity. |
| **Grade3）** | Severe or medically important, but not critical.  Need in-patient treatment or extension of hospitalization.  Incapacity / inoperability. Restriction of self care ADL**. |
| **Grade4** | Life-threatening. Need emergency treatment. |
| **Grade5** | Death caused by the adverse event. |

* instrumental ADL

：preparation for meal, daily shopping, telephone use, management of money, etc.

** self care ADL

：bathing, changing, eating, toileting, and ingestion. Not bedridden.

**^2)^ Definition of seriousness**

| ① to be fatal  ② life-threatening  ③ to need in-patient treatment or extension of hospitalization   1. to be a permanent or salient disability / incapacity 2. inducing congenital abnormality in offsprings |
| --- |

Excluding events: hospitalization appointed by the study procedure, hospitalization for a therapy or an examination scheduled prior to the study registration, and hospitalization except for a treatment / examination of adverse events.

**^3)^ Definition of predictivity**

| **○ Unpredictable (Unknown)**  Those of which tendency of the expression number, frequency, and condition of a concerned event can not predict from the official documents (instruction sheet, reports, etc.) of the relevant medicine/appalatus.   - **Predictable (Known)**   Those of which tendency of the expression number, frequency, and condition of a concerned event can predict from the official documents (instruction sheet, reports, etc.) of the relevant medicine/appalatus. |
| --- |

# Description of adverse events

Investigators etc. must describe in the CRF about name, severity (seriousness, non-seriousness), reason of desiding seriousness, date of onset and prognosis, treatment, outcome (recovered, remission, recovered with sequera, not recovered, or death), relationship with the relevant medicine/appalatus, comments (causality and reason of decision) regarding all adverse events.

- 1. The name of adverse event must be written with diagnosis name/ disease name in the CRF. If diagnosis name/ disease name can not be specified or investigators etc. decide appropriate not to describe diagnosis name/ disease name, clinical condition or symptoms (including abnormal lab data) can be alternatively written in the CRF.
  2. Describe the treatments (yes/no) for adverse events of the participant.

③ Describe the outcome of adverse events.

| **Outcome** | **Description** |
| --- | --- |
| Recovered | The adverse event is eliminated, or it returns to original. |
| Remission | Although the adverse event is not completely recovered, it is almost eliminated or returns to almost original. |
| Recovered with sequera | Although the adverse event is returned to original, a sequera remains. |
| Not recovered | The adverse event is ongoing. |
| Death | The adverse event causes death. |

Definition of adverse events:

All undesirable or unexpected diseases and injury or symptoms (including lab data abnormality) regardless of causality to the study. Collection of adverse events will be performed from registration to the end or termination of observation of each participant.

# Response to the serious adverse event / incidents (including adverse events to be reported to head of the research institute)

# Response to the adverse event / incident

1. In case of the adverse events / incidents, investigators etc. must deal with them appropriately. Investigators etc. must investigate their cause with extreme care of the participant.
2. Investigators etc. must continue observation and check the outcome of symptoms and lab data abnormality until they are eliminated or return to original or are diagnosed normal as long as possible.
3. If the adverse event / incident is not recovered or irreversible at the end of the study, or if investigators etc. decide it is not necessary to chase, investigators etc. can cease the observation at the end of the study and describe the reason why it is decided as no-need to chase in the comment field of the CRF.

# Report to head of the research institute and principal investigator (research director)

1. Principal investigator of the research institute must report the serious adverse event / incident within a period described below. The report must use a format “**(No 9) Document regarding serious adverse events**”.
2. In case of multi-institutional joint research, principal investigator of the research institute must report the serious adverse event / incident within a period described below. The report must use a format “**(No 1) Document regarding serious adverse events**”.
3. If the other facility is the representative institute, response may follow a formula of the representative institute.

**Necessity and period of report to head of the research institute and principal investigator (research director)**

**Causality no**

**Causality yes**

|  | **Grade1/2/3** | | | **Grade4** | | **Grade5** | | **Others, medically important condition** |
| --- | --- | --- | --- | --- | --- | --- | --- | --- |
|  | **Predictable (Known)** | **Unpredictable (Unknown)** | | **Predictable (Known)** | **Unpredictable (Unknown)** | **Predictable (Known)** | **Unpredictable (Unknown)** |  |
|  | **hospitalization**  **yes／no** | **hospitalization**  **no** | **hospitalization**  **yes** |  |  |  |  |  |
|  | Reoprt  necessary | Reoprt  unnecessary | Initial report:  within 10d  Additional report: occasionaly | 1^st^ report: within 72h  2^nd^ report: within 7d  Additional report: occasionaly | | | |  |
|  | Reoprt  unnecessary | Reoprt  unnecessary | Initial report:  within 10d*  Additional report: occasionaly* | 1^st^ report: within 72h*  2^nd^ report: within 7d*  Additional report: occasionaly* | | | |  |

* during treatment or within 30 days from the last protocol treatment

# Report to joint research institution

1. In case of multi-institutional joint research, a principal investigator (research director) must report the fact described below to a principal investigator of the research institute where the adverse event / incident was occurred and of the joint research institutes, and request to report to head of the institute and ethical committee.

| - 1. A report of serious adverse event |
| --- |

1. A principal investigator of the research institute takes an action of a nessesary protocol following the head of research institute.

# In case of invasive study, describe the existence and contents of compensation for health damage caused by the research

If health damage is occurred on a participant by the research procedure, the institute and a principal investigator must take a nessesary action. Medical cost for the treatment of health damage will be paied from own health insurlance, and there is no financial compensation.

# When there is a possibility that important knowledge such as health of a research participant and genetic characteristics that can be passed down to descendants may be obtained with the implementation of the research, including incidental findings

There is a possibility of finding any critical diseases, such as brain tumore, brain absess, brain hemorrhage, brain infarction, brain artery aneurysm, arteriovenous malformation etc., by analysis of MRI data. Also, neuropsychological examination may occasionaly find cognitive decline.

Basically, these obtained data will not inform to participants. But, a participant can express his/her request about knowing information of any abnormality found in examinations on the form of informed consent. If a participant requested and if any of such abnormality will be found, an investigator will inform the participant about to ask a doctor.

# Potential use of samples and information for future research that is not identified at the time of consent / A possibility of providing it to other research institutions

The data obtained in this study are expected to be used in another or future researches approved by the Ethics Committee. Confirm and follow the intention at the time of participation in the research on whether or not the results can be diverted. If the research to be diverted has not already been approved by the Ethics Committee, apply again to the Ethics Committee.

# How to publish information about the study

# Registration of research outlines and results

A study responsibility person registers the outline of the research in the public database (UMIN), changes the research plan, and updates it appropriately according to the progress of the research.

Items, including human rights of participants, human rights of researchers etc., matters to be kept private for the protection of intellectual property, and matters approved by the head of a research institution after receiving the opinion of the Ethics Committee, shall be kept private, because it would significantly interfere with research from the viewpoint of protecting personal information.

# Publication of research results

After the end of the study, the research responsibility person shall take measures to protect the personal information of the participants and publish the results of the study in scientific journals etc. without delay.

When the results are finally announced, they will be reported to the head of the research institution without delay.

# Method of storage and disposal of samples, information, etc.

# Strage

| **Samples and information etc. to be stored** | **Retention period** |
| --- | --- |
| - study samples | Discard immediately after the study finished. |
| ○ personal information obtained in the study (medical record, lab data, case report, etc.）  ○ Record of providing information and correspondence table  ○ study records, documents | 5 years from the end of study / 3 years from publication  （whichever longer） |

# Disporsal

A principal investigator must anonymize all data obtained from human prior to disporsal.

# What and how to report to the head of a research institute

The research director shall report the following to the head of a research institute in the prescribed form.

- Report facts that impair ethical validity and scientific rationality.
- Report when facts or information that impairs the appropriateness of the implementation of the research or the trust of the research results or information that may be damaged are obtained.
- Report the progress of research and the status of adverse events
- Report the status of management of samples and information acquired from the human body.
- Report the end of the research and the outline of the study results.

# Modification of the study protocol

When changing the research plan, the research director obtains the approval of the head of a research institution after review by the Ethics Committee.

1. **Amendment**

Changes of the study protocol those may increase a risk of participants or may influence the primary outcome. Those changes need approval of each institute. Cases are described below:

- 1. Changes those may increase participants’ burden (increase of blood tests or invasive examinations)
  2. Change of the excluding criteria etc. because of serious adverse events
  3. Change of assessing method of efficacy and safety
  4. Change of participants number

1. **Revision**

Changes of the study protocol those do not increase a risk of participants and do not influence the primary outcome. Those changes need approval of each institute. Cases are described below:

- 1. Changes those do not increase participants’ burden (Change of examination timing)
  2. Change of study period
  3. Change of investigators

1. **Memorandum**

Supplemental explanation sheet for reducing valiation of literal interpretation or inducing specific attention, instead of the change in the study protocol.

# Research implementation system

# Name of research institution, name of research director

Research Director: Yasuyuki Taki, Professor

Smart Aging Research Center, Tohoku University

# Name and role of research allotment person, etc.

(1) Co-investigator: Taizen Nakase, Smart Aging Research Center, Tohoku University

(2) Research co-investigators: Sayaka Makabe, Maiko Chiba, Aimi Abe, Izumi Matsudaira, Smart Aging Research Center, Tohoku University

Kouki Kobayashi, Hikari Iki, Haruka Asaoka, Yasuko Tatewaki, Department of Aging Research & Geriatric Medicine, IDAC, Tohoku University

(3) Materials manager: Taizen Nakase, Smart Aging Research Center, Tohoku University

(4) Data investigator: Taizen Nakase, Smart Aging Research Center, Tohoku University

# Research Secretariat, Statistical Analysis

(1) Research Office: Taizen Nakase, Smart Aging Research Center, Tohoku University

(2) Person in charge of statistical analysis: Taizen Nakase, Smart Aging Research Center, Tohoku University

(3) Data administrator: Taizen Nakase, Smart Aging Research Center, Tohoku University

(4) Data manager: Tatsushi Mutoh, Department of Aging Research & Geriatric Medicine, IDAC, Tohoku University

# Joint Research Institutes

1. Joint research institute: Yes (the name of the institution can be identified)

○ When the name of the institution can be identified

・Organization name: Ohki Pharmaceutical Co., Ltd.

・Names of research managers, etc.: Hirokazu Kawamoto

# Contact point for inquiries about research

(1) How to register the research subjects (participants): Taizen Nakase, Smart Aging Research Center, TohokuUniversity, 022-717-8824

(2) How to respond in the event of an adverse event: Taizen Nakase, Smart Aging Research Center, TohokuUniversity, 022-717-8824

# References

1. Moulton PL, Boyko LN, Fitzpatrick JL, Petros TV. The effect of Ginkgo biloba on memory in healthy male volunteers. Physiol Behav. 2001;73(4):659-65.
2. Mix JA, Crews Jr WD. An examination of the efficacy of Ginkgo biloba extract EGb761 on the neuropsychologic functioning of cognitively intact older adults. J Altern Comp Med 2000;6:219-229.
3. Dodge HH, et al. A randomized placebo-controlled trial of Ginkgo biloba for the prevention of cognitive decline. Neurology 2008;70(19):1809-1817.
4. Chen F, et al. Systemic and cerebral exposure to and pharmacokinetics of flavonols and terpenelactones after dosing standardized Gingko biloba leaf extracts to rats via different routes of administration. Br J Pharmacol 2013;170:440-457.
5. Wu Y, Sun J, George J, Ye H, Cui Z, Li Z, et al. Study of neuroprotective function of Ginkgo biloba extract (EGb761) derived-flavonoid monomers using a three-dimensional stem cell-derived neural model. Biotechnol Prog. 2016;32(3):735-44.
6. Wu Y, Wu Z, Butko P, Christen Y, Lambert MP, Klein WL, et al. Amyloid-beta-induced pathological behaviors are suppressed by Ginkgo biloba extract EGb 761 and ginkgolides in transgenic Caenorhabditis elegans. J Neurosci. 2006;26(50):13102-13.
7. Iwamoto K, et al. Mixing Ginkgo biloba extract with sesame extract and turmeric oil increases bioavailability of ginkgolide A in mice brain. J Oleo Sci 2019;68(9):923-930.
8. Hashiguchi M, Ohta Y, Shimizu M, Maruyama J, Mochizuki M. Meta-analysis of the efficacy and safety of Ginkgo biloba extract for the treatment of dementia. J Pharm Health Care Sci. 2015;1:14.
9. Zhang HF, et al. An overview of systematic reviews of Gingko biloba extracts for mild cognitive impairment and dementia. Front Aging Neurosci 2016;8:276.
10. Jiang L, Su L, Cui H, Ren J, Li C. Ginkgo biloba extract for dementia: a systematic review. Shanghai Arch Psychiatry. 2013;25(1):10-21..
