# Supplementary material for: Efficacy of a mixture of Ginkgo biloba, sesame, and turmeric on cognitive function in healthy adults: Study protocol for a randomized, double-blind, placebo-controlled trial
Source: PLoS One. 2023 Mar 15;18(3):e0280549. doi: 10.1371/journal.pone.0280549 (PMC10016651; doi:10.1371/journal.pone.0280549)
Supplement: S2 File — (DOCX) [file pone.0280549.s003.docx]

第9版　2018年8月28日

| **第Ⅲ種**  **研究の名称**：  健常人を対象としたイチョウ葉エキスおよびその吸収促進物質併用摂取による認知遂行機能への影響  （臨床試験登録番号：UMIN000043494）  研究責任者  瀧　靖之　（教授）  東北大学加齢医学研究所スマート・エイジング学際重点研究センター  臨床加齢医学研究分野  〒980-8575 仙台市青葉区星陵町４－１  TEL 022-717-8559　 FAX 022-717-8560  E-mail yasuyuki.taki.c7@tohoku.ac.jp  研究事務局  中瀬　泰然　（講師）  東北大学加齢医学研究所スマート・エイジング学際重点研究センター  臨床加齢医学研究分野  〒 980-8575 仙台市青葉区星陵町４－１  TEL 022-717-8559　 FAX 022-717-8560  E-mail taizen.nakase.a4@tohoku.ac.jp  2021年　8月　7日　作成（第6版） |
| --- |

目次

[1. 目的 5](#_Toc122877554)

[2. 背景と研究計画の根拠 5](#_Toc122877555)

[2.1. 背景 5](#_Toc122877556)

[2.2. 研究の科学的合理性の根拠 5](#_Toc122877557)

[3. 研究対象者の選定方針 6](#_Toc122877558)

[3.1. 研究対象者の母集団 6](#_Toc122877559)

[3.2. 適格基準 6](#_Toc122877560)

[3.3. 除外基準 6](#_Toc122877561)

[4. 予定症例数、設定根拠 6](#_Toc122877562)

[4.1. 予定症例数 6](#_Toc122877563)

[4.2. 設定根拠 6](#_Toc122877564)

[5. 統計解析 7](#_Toc122877565)

[5.1. 統計解析の方法 7](#_Toc122877566)

[6. 研究の方法、期間 7](#_Toc122877567)

[6.1. 研究デザイン 7](#_Toc122877568)

[6.2. 研究方法 7](#_Toc122877569)

[6.3. 研究期間 10](#_Toc122877570)

[7. 評価項目・方法 10](#_Toc122877571)

[7.1. 主要評価項目 10](#_Toc122877572)

[7.2. 副次的評価項目 10](#_Toc122877573)

[8. データの管理方法、自己点検の方法 10](#_Toc122877574)

[8.1. 症例記録（Case Report Form：CRF）の作成 10](#_Toc122877575)

[8.2. CRFの自己点検 11](#_Toc122877576)

[8.3. CRFの送付及び保管 11](#_Toc122877577)

[8.4. CRFの修正手順 11](#_Toc122877578)

[9. 研究の資金源等、研究機関の研究に係る利益相反及び個人の収益等、研究者等の研究に係る利益相反に関する状況 11](#_Toc122877579)

[10. 業務内容、委託先の監督方法 11](#_Toc122877580)

[11. 個人情報等の取扱い 12](#_Toc122877581)

[11.1. 個人情報の利用目的 12](#_Toc122877582)

[11.2. 利用方法（匿名化の方法） 12](#_Toc122877583)

[11.3. 安全管理責任体制（個人情報の安全管理措置） 13](#_Toc122877584)

[12. インフォームド・コンセントを受ける手続 13](#_Toc122877585)

[12.1. 研究対象者への説明 13](#_Toc122877586)

[12.2. 同意 14](#_Toc122877587)

[13. 代諾者等からインフォームド・コンセントを受ける場合の手続 14](#_Toc122877588)

[14. インフォームド・アセントを得る場合の手続 14](#_Toc122877589)

[15. 情報公開の手続 14](#_Toc122877590)

[16. 試料・情報の授受に関する記録の作成・保管 14](#_Toc122877591)

[17. 研究対象者に生じる負担、予測されるリスク（起こりうる有害事象を含む）・利益、これらの総合的評価、負担・リスクを最小化する対策 14](#_Toc122877592)

[18. 研究対象者等、その関係者からの相談等への対応 16](#_Toc122877593)

[19. 研究対象者等に経済的負担または謝礼がある場合、その旨、その内容 16](#_Toc122877594)

[20. 有害事象の評価 16](#_Toc122877595)

[20.1. 情報の入手 16](#_Toc122877596)

[20.2. 有害事象の記載 18](#_Toc122877597)

[21. 重篤な有害事象／不具合発生時の対応（研究機関の長に報告する有害事象範囲を含む） 18](#_Toc122877598)

[21.1. 有害事象／不具合発生時の対応 18](#_Toc122877599)

[21.2. 研究機関の長、研究責任者（研究代表者）への報告 19](#_Toc122877600)

[21.3. 共同研究機関への報告 19](#_Toc122877601)

[22. 侵襲を伴う研究の場合、研究によって生じた健康被害に対する補償の有無、内容 19](#_Toc122877602)

[23. 研究の実施に伴い、研究対象者の健康、子孫に受け継がれ得る遺伝的特徴等、重要な知見が得られる可能性がある場合、研究対象者に係る研究結果（偶発的所見を含む）の取扱い 20](#_Toc122877603)

[24. 試料・情報が同意を受ける時点では特定されない将来の研究のために用いられる可能性／他の研究機関に提供する可能性がある場合、その旨と同意を受ける時点において想定される内容 20](#_Toc122877604)

[25. 研究に関する情報公開の方法 20](#_Toc122877605)

[25.1. 研究の概要及び結果の登録 20](#_Toc122877606)

[25.2. 研究結果の公表 20](#_Toc122877607)

[26. 試料・情報等の保存・廃棄の方法 20](#_Toc122877608)

[26.1. 保存 20](#_Toc122877609)

[26.2. 廃棄 21](#_Toc122877610)

[27. 研究機関の長への報告内容、方法 21](#_Toc122877611)

[28. 研究計画書の変更 21](#_Toc122877612)

[29. 研究の実施体制 22](#_Toc122877613)

[29.1. 研究機関の名称、研究責任者の氏名 22](#_Toc122877614)

[29.2. 研究分担者等の氏名・役割 22](#_Toc122877615)

[29.3. 研究事務局、統計解析 22](#_Toc122877616)

[29.4. 共同研究機関 22](#_Toc122877617)

[29.5. 研究に関する問合せ窓口 22](#_Toc122877618)

[30. 引用文献 22](#_Toc122877619)

[31. Appendix 23](#_Toc122877620)

概要

- 1. **
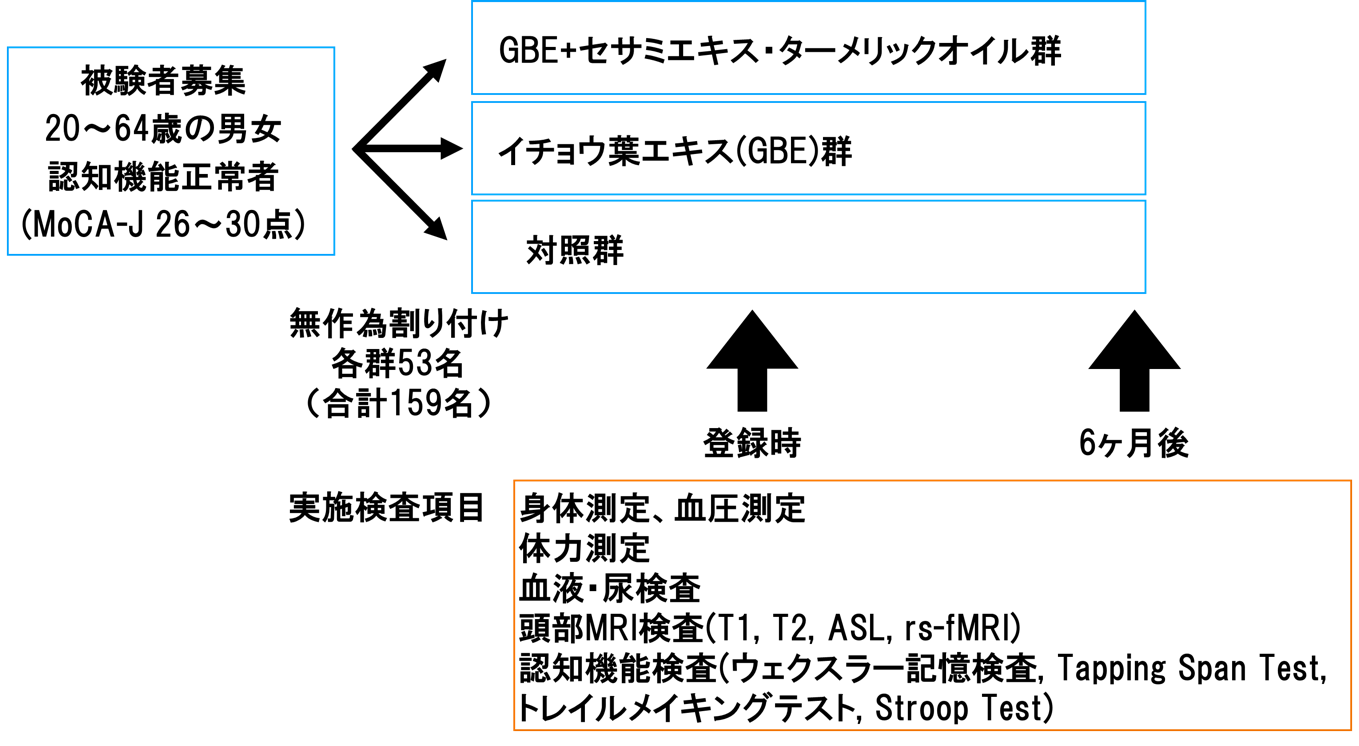
シェーマ**
  2. **目的**

イチョウ葉エキス（GBE）は、記憶力や集中力の向上が謳われるサプリメントとして市販されている。しかしイチョウ葉エキスの健常者に対する認知機能への影響については一定した見解は得られていない。本研究では健常成人を対象に、摂取イチョウ葉エキスの中枢神経系へのバイオアベイラビリティー向上による認知遂行機能への影響を検討する。さらに、この背景となる脳血流および神経細胞活動への影響を頭部MRI装置を用いて検証する。

- 1. **対象**

１）適格基準

一般から募集した20〜64 歳の男女から参加者を募る。認知機能が正常である者を対象とし、すべての研究対象者にMoCA-Jにてスクリーニングを行い、26点以上を適格基準とする。試験参加について本人から文書で同意が得られている者とする。

２）除外基準

登録時に以下の項目のいずれかに該当する場合は本研究の対象としない。

1. 重度の脳神経疾患の既往のある患者
2. 臨床的に問題となる出血症状のある患者
3. 抗血栓薬内服中の患者
4. クレアチニンクリアランス 15 mL/min未満の患者
5. インフォームドコンセントが得られない者
6. 研究担当医師が研究対象として不適当と判断した者
7. ペースメーカーや冠動脈ステント、人工関節などの体内金属留置患者
8. 閉所恐怖症
9. 糖尿病患者
10. 抑うつ患者
11. 重度の視覚障害、聴覚障害を有する患者
12. 消化器疾患、代謝性疾患を有する患者
13. 精神病または精神症状を合併し試験参加が困難と判断される研究対象者
14. 何らかのサプリメントを常用している者
15. 妊娠している方、授乳中の方
16. イチョウ葉エキスやごま、ターメリック（ウコン）に対してアレルギーのある方
    1. **予定症例数、研究期間**
17. 予定症例数：159例（対照群53例、GBE単剤群53例、GBE/MST配合剤群53例）、無作為割り付け
18. 研究期間：2021年4月（倫理委員会承認後）～2022年12月

（登録期間：～2022年2月、追跡期間：～2022年8月）

- 1. **問合せ先**

1. 適格基準、介入変更基準等、臨床的判断を要するもの：

東北大学加齢医学研究所スマート・エイジング学際重点研究センター

臨床加齢医学研究分野　　　中瀬泰然

〒 980-8575 仙台市青葉区星陵町４－１

TEL 022-717-8559 FAX 022-717-8560

1. 登録手順、記録用紙（CRF）記入等：

東北大学加齢医学研究所スマート・エイジング学際重点研究センター

臨床加齢医学研究分野　　中瀬泰然

〒 980-8575 仙台市青葉区星陵町４－１

TEL 022-717-8559 FAX 022-717-8560

# 目的

イチョウ葉エキス（GBE）の中枢神経系移行性を高めたセサミエキス・ターメリックオイル（MST）との配合剤による認知遂行機能への影響を明らかにする。

# 背景と研究計画の根拠

# 背景

イチョウ葉エキス (GBE: ginkgo biloba extract) は健常者に対するサプリメントとして市販され、その宣伝文句として記憶力や集中力の向上、アルツハイマー病の症状緩和などが謳われている。しかし科学的根拠としては、健常人に対して６週間の投与で実行機能に変化が見られたという報告^1)^や、85歳以上の健康高齢者において42カ月間の内服でアドヒアランスが良かった人のみ認知機能への影響が認められたという報告^2)^、20歳代の健康成人による５日間の内服では認知機能に変化を認めなかったという報告^3)^などがあり、その影響について定まった見解は得られていない。また、GBEに含まれる機能性成分のうち、フラボノールは脳実質でなく血漿中に蓄積されるのに対してテルペンラクトンは脳実質への透過性を示すが速やかに代謝されるなど、成分により体内動態も異なることが報告されている^4)^。

ゴマやゴマ油は古くから健康食品として用いられている。そのゴマより抽出されたセサミエキスにはsesaminとsesamolinが含まれており、基礎研究ではいずれも神経活性化に寄与することが報告されている^5)^。また、ターメリックオイルについても、その主成分であるα-およびβ-turmeroneが細胞活性化への影響があることが知られている^6)^。そして、セサミエキスはターメリックオイルとの同時投与によりバイオアベイラビリティーが改善するとの報告がある^7)^。

近年、大木製薬と近畿大学の共同研究で、GBEとセサミエキス・ターメリックオイル配合剤（MST: mixture of sesame extract and turmeric oil）を同時に投与することでGBEの中枢神経系への移行性が高まることが明らかになった^8)^。そこで今回、当分野と大木製薬との産学連携研究を企画しGBE単独投与とMST配合剤投与との間において認知機能への影響が異なるか観察することで、認知機能に関わるメカニズムのさらなる解明を目指す研究を立案した。

# 研究の科学的合理性の根拠

これまでの研究から、GBEの1日投与量は120mgと240mgで副作用による脱落率に差異を認めなかったと報告されている。また介入期間も12週間、22−24週間ともに安全性、脱落率に差異を認めなかった。

また、健常者に対するGBEの影響を見た研究では、実行機能への影響が見られたという報告^9)^や内服でアドヒアランスが良かった人のみ認知機能の変化が認められたという報告^2)^、認知機能には変化を認めなかったという報告^3)^などが様々な結果があり議論の余地が残っている。

さらにGBEのバイオアベイラビリティーについては、GBEに含まれる有効成分のうちフラボノールは中枢神経で検出できずテルペンラクトンのみ検出できるが、その濃度は非常に低いと報告されている^4)^。これに対してマウスを用いた実験で、GBEをMSTと混合投与することで血清中および脳組織ともにおいてテルペンラクトンの有意な濃度上昇が得られている^8)^。

　本研究の実施の適否に関し、倫理的、科学的妥当性の観点から倫理委員会の審査を受け、研究機関の長による承認を得る。

# 研究対象者の選定方針

# 研究対象者の母集団

母集団は健常成人。

# 適格基準

1. 認知機能正常の健常成人で、本人より研究参加への同意が得られている者。
2. 認知機能　Montriol Cognitive Assesment Japanese version (MoCA-J)：26点以上。認知症疾患診療ガイドライン^9)^ によると軽度認知機能障害を除外するにはMini Mental State Examination (MMSE) や長谷川式認知機能検査では十分でないためMoCA-Jが推奨されている。
3. 年齢：20歳以上、64歳以下（登録時）。過去の報告より、20歳代の健常成人に対して５日間の投与でプラセボとの間に変化を認めず^3)^、55歳から86歳の健常者に対して６週間の投与で遂行機能に変化が見られている^9)^。健常成人において認知機能は年齢の影響が大きいと考えられ、また認知症やその他の合併疾患からの影響を避ける目的も含めて、対象年齢を20歳から64歳と設定した。
4. 性別：男女問わない。

# 除外基準

登録時に以下の項目のいずれかに該当する場合は本研究の対象としない。

1. 重度の脳神経疾患の既往のある患者
2. 臨床的に問題となる出血症状のある患者
3. 抗血栓薬内服中の患者
4. 研究担当医師が研究対象として不適当と判断した者
5. ペースメーカーや冠動脈ステント、人工関節などの体内金属留置患者
6. 閉所恐怖症
7. 糖尿病患者
8. 抑うつ患者
9. 重度の視覚障害、聴覚障害を有する患者
10. 何らかのサプリメントを常用している者
11. クレアチニンクリアランス 15 mL/min未満の患者
12. 消化器疾患、代謝性疾患を有する患者
13. 精神病または精神症状を合併し試験参加が困難と判断される研究対象者
14. インフォームドコンセントが得られない患者
15. 妊娠している方、授乳中の方
16. イチョウ葉エキスやごま、ターメリック（ウコン）に対してアレルギーのある方
17. その他、研究責任医師または研究分担医師が試験参加を困難と判断した者

# 予定症例数、設定根拠

# 予定症例数

予定症例数：159例（対照群53例、GBE単剤群53例、GBE/MST配合剤群53例）

# 設定根拠

本研究における主要評価項目は、介入24週間後のウェクスラー記憶検査（WMS）結果の変化を検討することである。WMSは正常平均を100としたとき標準偏差が15を示すように設計されている。本研究におけるWMSスコア標準偏差の変化を1％と仮定すると平均の差は6となる。Cohenの式に従い効果量が0.4、有意水準（α）を0.05、検出力（1-β）を80％として計算すると必要症例数は44例以上となる。そこで本研究では20％の脱落率を想定して各群53例と設定した。

# 統計解析

# 統計解析の方法

全ての被験者を解析の対象とする。ただし、画像の撮像条件等で適切なデータが得られなかったものは除外する。

1. GBE/MST配合剤摂取に関連する認知遂行機能の検討

各種認知心理検査のスコアを介入前後で比較し、認知遂行機能の変化が見られた機能特性とGBE単剤摂取群とGBE/MST配合剤摂取群との関連性を評価する。各々の得点の平均値の比較はt検定を用いる。

1. GBE/MST配合剤摂取による脳血流の変化の検討

MRI灌流画像としてarterial spin labeling (ASL) 法により全脳または領域ごとの平均信号強度を測定し、介入前後でペアt検定を用いて比較する。さらに介入前後の、解剖学的に標準化された脳血流分布mapを、SPM12を用いてペアt検定によってボクセルごとに比較し、有意な変化を示す領域があるか検討する。

# 研究の方法、期間

# 研究デザイン

1. **研究デザイン**
   1. **対照**

賦形剤のみのカプセル

- 1. **盲検化**

二重盲検比較

- 1. **比較方法**

平行群間比較、ランダム化比較

1. **研究デザインの設定根拠**

本試験の主たる研究仮説は「介入開始6か月後の認知心理検査結果の変化と頭部MRIでの脳血流の分布の変化」である。本研究で用いる試験製品はMSTが配合されたGBEの検討であり、過去に報告されているGBEのみの研究結果と比較するためにMST配合製品とGBEのみの製品およびプラセボとの３群間で比較検討を行う二重盲検ランダム化比較試験とした。

# 研究方法

1. **試験薬**

〇介入製品

・GBE/MST配合製剤

イチョウ葉エキス、セサミ・ターメリックオイル配合カプセル（イチョウ葉エキス120mg、ヒハツ抽出物120mg、セサミ・ターメリックオイル120mg、賦形剤）：研究にはパッケージ印刷のないものを使用する。

・GBE製剤

イチョウ葉エキスカプセル（イチョウ葉エキス120mg、賦形剤）：研究にはパッケージ印刷のないものを使用する。

・本試験製品は、大木製薬株式会社より販売されている製品『ブレインアシスト』および他社製品『イチョウ葉エキス錠』と同等の製品である。ブレインアシストはすでに10万個以上の販売実績を持っており、イチョウ葉エキス製剤は多数の会社から長年にわたり販売されている。

・本試験製品の原材料配合、製造場所、衛生管理基準は、市販品と同レベルで製造されている。

・被験者および検査者へのバイアスをなくすため、本研究ではパッケージ印刷のない銀無地の包装を行う。

『GBE/MST配合製剤』

・試験製品の内容量　　60粒／袋

・原材料　イチョウ葉エキス末（スイス製造）、ヒハツエキス（ごま油、ヒハツエキス）、還元水飴、ＤＨＡ含有精製魚油／ゼラチン、香辛料抽出物、グリセリン、グリセリン脂肪酸エステル、ミツロウ、カラメル色素、植物レシチン（大豆由来）

・栄養成分　１日２粒（800ｍｇ）あたり

エネルギー・・・4kcal、たんぱく質・・・0.2ｇ、脂質・・・0.3ｇ、炭水化物・・・0.2ｇ、食塩相当量・・・0.0008ｇ

『GBE製剤』

・試験製品の内容量　　60粒／袋

・原材料　食用油脂、イチョウ葉エキス末（スイス製造）、還元水飴/ゼラチン、グリセリンエステル、トリコエノールレシチン（大豆由来）

・栄養成分　１日２粒（800ｍｇ）あたり

エネルギー・・・5kcal、たんぱく質・・・0.2ｇ、脂質・・・0.4ｇ、炭水化物・・・0.2ｇ、食塩相当量・・・0.0009ｇ

〇対照製品

・機能性関与成分を含まない賦形剤のみとする。『GBE/MST配合製剤』、『GBE製剤』は共にカプセル製剤であり、形状、色、味ともに似ている。そのため、対照製品（プラセボ）もこの２製剤に形状、色、味とも似せた製品とする。

・原材料：還元水飴、ＤＨＡ含有精製魚油／ゼラチン、香辛料抽出物、グリセリン、グリセリン脂肪酸エステル、ミツロウ、カラメル色素、植物レシチン（大豆由来）。

・栄養成分　１日２粒あたり

エネルギー・・・5kcal、たんぱく質・・・0.2ｇ、脂質・・・0.5ｇ、炭水化物・・・0.1ｇ、食塩相当量・・・0.009ｇ。

1. **用法・用量／摂取レベル**

GBE/MST群：GBE/MST配合製剤1日１回２カプセルを水またはお湯で服用する。

GBE群：GBE製剤１日1回２カプセルを水またはお湯で服用する。

プラセボ群：プラセボ１日1回２カプセルを水またはお湯で服用する。

1. **中止・完了基準**

GBEの有害事象として、頭痛、めまい、耳鳴、下痢、嘔気、消化管症状、血圧上昇、口渇、息切れ、上気道感染、胸痛、徐脈などが報告されている。しかし興味深いことに、プラセボに対してGBEの方が有害事象発現率が少なくなっているという報告もある^10)^。

Grade 3の有害事象の出現または有害事象による被験者からの拒否があった際には研究を中止する。

・有害事象：担当医判断または中止規定に従った有害事象による摂取中止。

・拒否（有害事象）：有害事象に関連する研究対象者拒否による摂取中止。

・拒否（その他）：転居による場合等、有害事象に関連しない研究対象者拒否による摂取中止。

・死亡：プロトコール治療中の死亡（因果関係の有無を問わない）。

・その他：上記以外の理由による中止。

1. **検査スケジュール**

**登録時**

　本人の書面による同意を確認

認知心理検査　東北大学病院または加齢医学研究所で実施

Montreal Cognitive Assesment 日本語版（26点以上）

**介入前**

・血液検査（採血量 約15ml）：加齢医学研究所または東北大学病院で実施

＜検査項目＞

血算：WBC（分画含む）、RBC、HGB、HCT、PLT

生化学：GOT、GPT、LDH、ALP、G-GTP、CPK、TP、ALB、UA、BUN、CRE、T-BIL、Na、K、CL、CA、MG、TC、TG、LDL-C、HDL-C、GLU

・尿検査：加齢医学研究所または東北大学病院で実施

　尿糖、尿蛋白（定性）、尿潜血、尿中白血球

・身体測定：加齢医学研究所または東北大学病院にて実施

身長、体重、血圧、両手の握力、2 Step Test、Functional Reach Testを行う。

（血液検査、尿検査、身体測定は、介入前後における栄養状態、肝機能、腎機能に変化がないこと、健康状態に問題がないことを確認する目的で行う。）

・MRI検査：加齢医学研究所または東北大学病院にて実施

　Philips社の3テスラMRIを用いて、頭部のArterial Spin Labeling画像、T1強調画像、T2強調画像、DTI (diffusion tensor imaging)、rs-fMRI（安静時機能的MRI）を撮像する。

・認知心理検査：加齢医学研究所または東北大学病院にて実施

MRI前にウェクスラー記憶検査を、MRI後にTrail Making Test-A, B、Stroop test、Tapping Span testを予定する。

**介入（24週間）後**　　＊ただし2週間の前後を許容する

　検査内容は介入前と同様とする。

検査日スケジュール

検査前日21時までに夕食を摂取、以後絶食とする

糖分を含まない水分は摂取可

朝食をとらずに9時来院

10時～11時　 検査前確認、採血、採尿、身体測定

11時～12時 認知心理検査①

12時～13時 昼食、休憩（食事は研究者が用意）

13時～14時 MRI検査、休憩

14時～15時　 認知心理検査②

# 研究期間

2021年6月（倫理委員会承認後）～2022年12月31日

# 評価項目・方法

# 主要評価項目

　24週間のGBE/MST配合剤経口投与により認知遂行機能に何らかの変化が見られるか評価するため、投与開始前と24週後とでウェクスラー記憶検査結果を比較する。

# 副次的評価項目

1．認知心理検査

　a) MoCA-J …認知機能スクリーニングに用いる

b) Trail Making Test-A, B　　…注意力検査に用いる

c) Stroop test　　…前頭葉機能の評価に用いる

d) Tapping Span test　　…視覚性記憶検査に用いる

　e) Digit Span test　　…言語性記憶検査に用いる

2．脳MRI検査

脳灌流画像：非侵襲的脳灌流画像であるArterial Spin Labeling法を用いてCBF mapを得る。SPMを用いてCBF mapを標準化し、解剖学的に標準化されたrCBF mapを作成する。

脳構造画像：SPM8(Statistical Parametric Mapping)やFSL(FMRIB Software Library)などの画像解析ソフトウェアを用いて、3D-T1強調画像の解剖学的標準化を行い、被験者毎の個人差を取り除く。各被験者の標準脳座標における「灰白質の存在確率マップ」、「白質の存在確率マップ」をそれぞれ作成する。DTI(diffusion tensor imaging), rs-fMRI（安静時機能的MRI）を撮像し、それぞれ解剖学的結合、機能的結合の評価を行う。

3．身体測定

　a)　身長、体重

　b)　体力の指標として、両手の握力、2 Step Test、Functional Reach Test を介入前後で測定する。

# データの管理方法、自己点検の方法

# 症例記録（Case Report Form：CRF）の作成

CRFの記載の記入及び訂正は研究者等（担当医）が行う。研究者等は各研究対象者の各観察・検査が終了後、速やかにCRFを作成する。

　誤登録・重複登録が判明した場合、速やかに研究事務局に連絡する。

# CRFの自己点検

1. 研究者等は、CRF内容と原資料（診療録、生データ等）の整合を確認する。
2. CRFと原資料に矛盾がある場合、その理由を説明する記録を作成する。
3. 研究機関の研究責任者または研究分担者は、作成されたCRFについてその内容を点検し、確認した上で記名・押印又は署名を行う。

# CRFの送付及び保管

研究機関の研究責任者は、作成したCRFを定められた手順にて原本を研究事務局に提出し、写しを保管する。提出先は下記とする。

**（CRFの提出先）**

東北大学加齢医学研究所スマート・エイジング学際重点研究センター

臨床加齢医学研究分野　（研究事務局）

〒 980-8575 仙台市青葉区星陵町４－１　TEL 022-717-8559 FAX 022-717-8560

# CRFの修正手順

CRFを訂正する場合、研究機関の研究責任者はCRFの変更又は修正の記録を定められた手順にて提出しその写しを保管する。

# 研究の資金源等、研究機関の研究に係る利益相反及び個人の収益等、研究者等の研究に係る利益相反に関する状況

本研究は、大木製薬株式会社および東北大学ナレッジキャスト(株)※との共同研究契約に基づき、大木製薬株式会社から受け入れた研究費および製剤を使用し、大木製薬株式会社が製造販売するサプリメント「ブレインアシスト」の認知機能への影響を検討する。

本研究における利益相反については、世界医師会ヘルシンキ宣言及び人を対象とする医学系研究に関する倫理指針（文部科学省、厚生労働省）において、研究対象者への資金提供、スポンサー、利益相反に関する十分な説明と研究計画書への記載が求められることを踏まえ、研究計画書及び研究対象者への同意説明文書にも記載するものとする。なお、利益相反マネジメント方法については各施設の基準に委ねる。東北大学の研究者等の利益相反は、東北大学利益相反マネジメント委員会が管理する。

資金提供者は研究対象者の登録やデータ管理・解析には一切関わらない。

※ 研究成果の社会活用促進を目的とすると東北大学の子会社。コンサルティング、人材育成を事業内容とする。

# 業務内容、委託先の監督方法

製品管理、被験者募集のためのチラシ作成、広報誌などへの取次は大木製薬が行う。

# 個人情報等の取扱い

# 個人情報の利用目的

研究の正しい結果を得るために、介入中だけではなく介入終了後も長期間にわたり研究対象者個人を特定して調査を行うこと、取得した情報を適切に管理することを目的として個人情報を利用する。

# 利用方法（匿名化の方法）

**（１）個人情報等の有無について**

| 種類 | | 定義 | 具体例 | 有無 |
| --- | --- | --- | --- | --- |
| 個人情報 | | ①情報単体で特定の個人を識別することができるもの | 氏名・顔画像等 | ■有  □無 |
|  |  | ②他の情報と照合することによって特定の個人を識別することができるもの | 対応表によって特定の個人を識別することができる他の情報と照合できるもの |  |
|  |  | ③個人識別符号が含まれるもの | ゲノムデータ　等 | □有  （具体的に：　　　）  ■無 |
|  | 要配慮個人情報 | 病歴、社会的身分、人種、信条、犯罪の経歴、犯罪により害を被った事実その他本人に対する不当な差別、偏見その他の不利益が生じないようにその取扱いに特に配慮を要する記述等が含まれる個人情報 | 診療録、レセプト、健診の結果、一部のゲノム情報^※^等 | ■有  （具体的に：検査結果）  □無 |

※　個人識別符号に該当するゲノムデータに単一遺伝子疾患、疾患へのかかりやすさ、治療薬の選択に関するものなどの解釈を付加し、医学的意味合いを持った「ゲノム情報」は、要配慮個人情報に該当する場合がある。

**（２）匿名化の有無**

■匿名化する（（3）へ）

□匿名化しない（理由：　　　　　　　）

□その他（具体的に：　例：行政機関/独立行政法人等個人情報保護法の規定の適用を受ける非識別加工情報とする。）

**（３）匿名化の種類及び方法**

■1）匿名化されている。（特定の個人を識別することができる対応表を本学で作成しているものに限る）

方法：研究対象者のデータや検体から氏名等の特定の個人を識別することができることとなる記述等を削り、代わりに新しく符号又は番号をつけて匿名化を行う研究対象者とこの符号（番号）を結びつける対応表を東北大学で作成し、個人情報管理者は外部に漏れないように厳重に保管する。

□2）匿名化されている（特定の個人を識別することができないものであって、対応表を他施設で保有しているものに限る。）

　方法：研究対象者のデータや検体から氏名等の特定の個人を識別することができることとなる記述等を削り、代わりに新しく符号又は番号をつけて匿名化を行う研究対象者とこの符号（番号）を結びつける

対応表を東北大学内で保有していない。（東北大学以外で対応表を保有しているが東北大学では保有していない）また、東北大学の他学部等で保有している場合も当てはまらない。

□3）匿名化されている（特定の個人を識別することができないものであって、対応表が作成されていないものに限る）

方法：研究対象者のデータや検体から氏名等の特定の個人を識別することができることとなる記述等を削り、代わりに新しく符号又は番号をつけて匿名化を行う研究対象者とこの符号（番号）を結びつける対応表は作成しない。（この研究において、全ての施設で対応表を作成していない）

□4）その他　（具体的に：　　　　）

# 安全管理責任体制（個人情報の安全管理措置）

東北大学における個人情報管理者

　・氏名：中瀬泰然

　・所属部局・所属分野：加齢医学研究所スマート・エイジング学際重点研究センター　臨床加齢医学研究分野

　・国家資格：医師

管理方法：紙・電子媒体での保存を行う。匿名化の対応表は研究データと別に厳重に保管する。

　以下の４点を行う。

　・物理的安全管理（データ管理PCは機能画像医学研究分野研究室内の保管庫にて鍵をかけて保管、記録媒体の持ち出し禁止等、盗難等・漏えい等の防止、個人データの削除及び機器、電子媒体等の廃棄）

　・技術的安全管理（データ管理PCへのアクセス制御、外部からの不正アクセス等の防止に対して不正ソフトウェア対策）

　・組織的安全管理（個人情報の取扱の制限と権限を研究責任者及び研究分担者に限定する）

　・人的安全管理（定期的に教育を受ける）

# インフォームド・コンセントを受ける手続

# 研究対象者への説明

研究者等は、登録前に研究機関の承認を得た説明文書を研究対象者に渡し、以下の内容を説明する。

**（説明文書記載事項）**

①研究の名称、研究実施について研究機関の長の許可を受けている旨

②研究機関、研究責任者

③研究の目的、意義

④研究の方法、期間

⑤研究対象者として選定された理由

⑥研究対象者に生じる負担並びに予測されるリスク、利益

⑦研究実施・継続に同意した場合も随時これを撤回できる旨

⑧研究実施・継続の不同意・同意撤回により研究対象者等が不利益な取扱いを受けない旨

⑨研究に関する情報公開の方法

⑩研究対象者等の求めに応じ他の研究対象者の個人情報等の保護や研究の独創性の確保に支障がない範囲内で研究計画書、研究の方法に関する資料入手・閲覧方法

⑪個人情報等の取扱い（匿名化する場合はその方法を含む）

⑫試料・情報の保存、廃棄の方法

⑬利益相反に関する状況（研究の資金源、起こり得る利害の衝突、研究者等の関連組織との関わり）

⑭研究対象者等及びその関係者からの相談等への対応

⑮研究対象者等に経済的負担・謝礼がある場合の内容

⑯研究の実施に伴う研究対象者の健康、子孫に受け継がれ得る遺伝的特徴等、重要な知見が得られる可能性がある場合、研究対象者に係る研究結果（偶発的所見を含む）の取扱い

⑰侵襲を伴う研究の場合、研究によって生じた健康被害に対する補償の有無、内容

⑱研究対象者から取得された試料・情報について、研究対象者等から同意を受ける時点では特定されない将来の研究のために用いられる可能性または他の研究機関に提供する可能性がある場合の同意を受ける時点において想定される内容

# 同意

研究についての説明を行い、十分に考える時間を与え、研究対象者が試験の内容をよく理解したことを確認した上で、試験への参加について依頼する。研究対象者本人が試験参加に同意した場合、同意文書に研究対象者本人による署名を得る。

同意文書は、原本を研究機関の研究責任者が保管し、写しを研究対象者本人に渡す。

# 代諾者等からインフォームド・コンセントを受ける場合の手続

代諾者は許可せず、必ず本人からの同意を得る。

# インフォームド・アセントを得る場合の手続

　該当なし。

# 情報公開の手続

該当なし。

# 試料・情報の授受に関する記録の作成・保管

該当しない。

# 研究対象者に生じる負担、予測されるリスク（起こりうる有害事象を含む）・利益、これらの総合的評価、負担・リスクを最小化する対策

1. **予測される利益**

取得された対象者の脳MRI画像は放射線科専門医による読影を行い、読影結果が回付されるため、対象者は現在の健康状態を示す一部の情報を知ることができる。

1. **予測される危険と不利益**

研究参加によって検査実施に関わる危険性と検査の実施にかかる時間的拘束および加齢医学研究所までの移動にかかる時間的拘束と交通費にかかる経済的不利益が生じる。

採血に関連して予測される危険と不利益

採血は健康診断で行われる範囲内の採血量、実施手順である。そのため、

1.穿刺部位からの出血が生じ、内出血のために皮下が青くなったりする。対策として採血後は5分間の圧迫止血を徹底する。

2.採血後の穿刺部にテープを貼るためテープかぶれを生じる可能性がある。対策として事前にテープにかぶれやすい人は申し出てもらう。通常のテープとは異なるビニールテープなどで対応する。

3.消毒のためのアルコール綿に対するアレルギー反応が生じる可能性がある。対策として、事前にアルコールに対するアレルギーの有無を聴取しておく。アルコールアレルギーのある人にはクロルヘキシジンによる消毒を行う。

4.痛みや緊張により血管迷走神経反射が起こり気分不良、意識消失などが生じる可能性がある。対策として、血管迷走神経反射の既往がある人には事前に申し出てもらう。採血前後は安静を保てる場所で過ごしてもらう。

5.採血による神経損傷が生じる可能性がある。採血に際して神経を損傷ししびれや痛みが持続する病態であり、軽度なものを含めると約6000回に1回起こると報告されている。解剖学的に完全に予防することはできないため、予防策として推奨されている手順に従い、通常外来における採血室で行うこととする。万が一、発生した場合は各自の健康保険により自己負担で医療機関を受診することで対応する。

MRI撮像に関連して予測される危険と不利益

1.MRI 撮像に伴い体内・体表の金属に起因する傷害が生じる可能性があるため、下記の対策を講じる。

・手術歴、体内金属の有無、ペースメーカー装着の有無について、事前に書面と問診とで複数回確認を行う。

・検査直前にも金属を身に付けていないことを確認する。

・検査中に不快を感じた場合には、いつでも検査を中断できることを予め伝えておく。

2. MRI の強力な磁場により金属物が飛来して、物理的な傷害を引き起こす可能性があるため、下記の対策を講じる。

・MRI 室には固定されていない金属物は決して搬入しない。

・実験を行う施設では誤って金属物が搬入される危険性を設備面・教育面で厳重に防いでおり、これらの安全対策に十分経験のあるスタッフが実験を厳重にコントロールする。

3. MR 画像解析において、脳腫瘍、脳膿瘍、脳出血、脳梗塞、脳血管動脈瘤、脳動静脈奇形などの緊急性を要する異常所見が発見される可能性がある。

これら異常所見が認められた場合は、参加者の事前の希望に応じて、医療機関への受診を勧める。

4. MRI 撮像に関わる騒音や磁場、温熱効果、閉鎖空間での体位固定に伴い気分不快を感じる可能性がある。

MRI 撮像時に生じる騒音による気分不快や、MRI で使用している強い磁場による「磁場酔い」と呼ばれる気分不快症状、MRI 撮像で発生する熱による体熱感を感じる場合があり、さらに狭い空間で動くことができないことによる不安感を感じることがある。

これらの対策として、撮像中は被験者の手にブザーを持ってもらい、気分が悪くなった際には押して知らせてもらうようにする。ブザーが押された場合には撮影を一旦中断して医師が被験者の状況を詳細に観察し、続行が難しい場合には検査を中止する。

GBEおよびGBE/MST摂取に関連して予想される危険と不利益

　イチョウ葉エキス、ゴマエキス、ターメリックは古くから食品として使用されており、安全性は確立されている。現在までに経口摂取による重篤な副作用の報告はなく、消化管症状、口渇などが報告されている。これら症状は摂取の中止により3､4日で改善が認められている。もし副反応が重大で研究を継続できない場合には研究への参加を中止することができる。

# 研究対象者等、その関係者からの相談等への対応

　研究全般に関する問合せ窓口（連絡先）

東北大学加齢医学研究所スマート・エイジング学際重点研究センター

臨床加齢医学研究分野　　　中瀬泰然

〒 980-8575 仙台市青葉区星陵町４－１

TEL 022-717-8559 FAX 022-717-8560

プライバシーポリシーに関する問合せ窓口（連絡先）

東北大学加齢医学研究所スマート・エイジング学際重点研究センター

臨床加齢医学研究分野　　　中瀬泰然

〒 980-8575 仙台市青葉区星陵町４－１

TEL 022-717-8559 FAX 022-717-8560

# 研究対象者等に経済的負担または謝礼がある場合、その旨、その内容

研究参加によって検査の実施にかかる時間的拘束だけではなく、自宅から加齢医学研究所までの移動にかかる時間的拘束と交通費にかかる経済的不利益が生じる。

- 介入に使用するGBE/MSTおよびGBE製品は大木製薬より提供を受けるため、被験者に費用負担は生じない。

MRI検査と認知心理検査に係る費用は企業との共同研究費で負担する。

二回にわたる検査を全て実施した参加者にのみ、謝礼として現金10,000円（介入前後の検査、各1日、日給5,000円として計算）を支払う予定である。介入期間中に参加を中止した被験者にはその一部を支払う。

# 有害事象の評価

# 情報の入手

1. 研究者等（担当医）は、重篤な有害事象／不具合が発現した場合、適切な処置を行い、研究機関の研究責任者に報告する。
2. 研究機関の研究責任者は、研究者等に以下を確認する。

**研究責任者による研究者等への確認事項**

| ①有害事象名／不具合名  ②重症度分類^1)^  ③重篤性^2)^ 、重篤と判断した理由  ④予測性（未知・既知）^3)^  ⑤介入（医薬品／医療機器）との因果関係  ⑥事象／不具合の経緯（発現日、経過、転帰等）  ⑦研究対象者の特定に関する情報（イニシャル、年齢、性別） |
| --- |

**^1)^重症度分類**

有害事象/有害反応の評価には「有害事象共通用語規準v4.0 日本語訳JCOG 版（NCI-CommonTerminology Criteria for Adverse Events v4.0（CTCAE v4.0）の日本語訳）」（以下、CTCAE v4.0-JCOG）を用いる。なお、CTCAE v4.0-JCOGのうち、臨床検査値の施設基準値でGrade が定義されている項目については、個々の医療機関における施設基準値の代わりに「JCOG 共用基準範囲」を用いる。「JCOG 共用基準範囲」の詳細はJCOG ウェブサイト（http://www.jcog.jp/doctor/tool/kijun.html）を参照すること。

NCI CTCAE分類に該当する項目がない場合、以下**「有害事象の重症度分類基準」**を参考に判定する。

| **重症度分類**  **（NCI CTCAE Grade）** | **基準** |
| --- | --- |
| **軽症　（Grade1）** | 症状がない、または軽度の症状がある。臨床所見または検査所見のみ。  治療を要さない。 |
| **中等症　（Grade2）** | 最小限/局所的/非侵襲的治療を要する。  年齢相応の身の回り以外の日常生活動作の制限^*^。 |
| **重症　（Grade3）** | 重症または医学的に重要であるが、ただちに生命を脅かすものではない。  入院または入院期間の延長を要する。  活動不能/動作不能。身の回りの日常生活動作の制限^**^。 |
| **最重症　（Grade4）** | 生命を脅かす。緊急の処置を要する。 |
| **死亡　（Grade5）** | 有害事象（AE）による死亡。 |

^*^身の回り以外の日常生活動作（instrumental ADL）

：食事の準備、日用品や衣類の買い物、電話の使用、金銭の管理等。

^**^身の回りの日常生活動作（self care ADL）

：入浴、着衣・脱衣、食事の摂取、トイレの使用、薬の服薬が可能で、寝たきりではない状態。

**^2)^重篤の定義**

| ①死に至るもの  ②生命を脅かすもの  ③治療のための入院又は入院期間の延長が必要となるもの  ④永続的又は顕著な障害・機能不全に陥るもの  ⑤子孫に先天異常を来すもの |
| --- |

研究計画書で規定する入院、研究前（同意取得前）より予定していた療法または検査を研究実施中に実施することのみを目的とした入院（予定手術や検査等）、有害事象に伴う治療・検査の目的以外の入院（健康診断等）は重篤な有害事象として取扱わない。

**^3)^予測性の定義**

| **○予測できない（未知）**  当該事象等の発現、あるいは発現数、発現頻度、発現条件等の発現傾向が当該医薬品／医療機器に関する公式文書（添付文書や論文等）から予測できないもの  **○予測できる（既知）**  当該事象等の発現、あるいは発現数、発現頻度、発現条件等の発現傾向が当該医薬品／医療機器に関する公式文書（同上）から予測できるもの |
| --- |

# 有害事象の記載

研究者等は、発現したすべての有害事象に関し、有害事象名、程度（重篤、非重篤）、重篤と判断した理由、発現日、転帰日、処置、転帰（回復、軽快、回復したが後遺症あり、未回復、死亡）、医薬品／医療機器との因果関係、コメント（因果関係と判定理由等）を症例報告書に記載する。

①有害事象名は、原則として診断名・疾患名（病名）で症例報告書に記載する。診断名・疾患名が特定できない場合や研究者等が診断名・疾患名としないことが妥当と判断した場合、臨床症状または徴候（臨床検査値異常を含む）を有害事象名として症例報告書に記載する。

②有害事象を治療のために研究対象者に対して取られた処置（あり／なし）を記載する。

③有害事象の転帰を記載する。

| **転帰の分類** | **解説** |
| --- | --- |
| **回復** | 有害事象が消失、または元の状態まで戻っている |
| **軽快** | 有害事象は完全に回復していないものの、ほぼ消失、またはほぼ元の状態に戻っている |
| **回復したが後遺症あり** | 有害事象は元の状態まで回復したものの、後遺症が残っている |
| **未回復** | 有害事象は継続中である |
| **死亡** | 有害事象の結果、死亡した |

本研究における有害事象の定義：

実施された研究との因果関係の有無を問わず、研究対象者に生じた全ての好ましくない又は意図しない傷病若しくはその徴候（臨床検査値の異常を含む。）とする。有害事象の収集は、研究への参加以降、個々の研究対象者の観察期間終了または中止時までの期間とする。

# 重篤な有害事象／不具合発生時の対応（研究機関の長に報告する有害事象範囲を含む）

# 有害事象／不具合発生時の対応

1. 研究者等は、有害事象／不具合が発現した場合、適切な処置を施し、研究対象者の安全確保に留意して原因究明に努める。
2. 研究者等は、発現した症状あるいは臨床検査値の異常変動について、原則として当該事象が消失または研究開始前の状態に回復するまで、または臨床上問題とならないと判断されるまで、可能な限り経過観察を継続し、その転帰を確認する。
3. 研究終了時に未回復の有害事象／不具合が非可逆的な事象の場合等、研究者等が追跡不要と判断した場合、研究対象者の研究終了時をもって追跡終了し、症例報告書のコメント欄に追跡不要と判断した理由を記載する。

# 研究機関の長、研究責任者（研究代表者）への報告

1. 研究機関の研究責任者は、重篤な有害事象／不具合の発現を知った時点から以下の期限内に研究機関の長に報告する。報告は、**「（様式第9号）重篤な有害事象に関する報告書」**を用いる。
2. 多施設共同研究の場合、研究機関の研究責任者は、重篤な有害事象／不具合の発現を知った時点から以下の期限内に研究責任者（研究代表者）に報告する。報告は、**「（参考書式１）重篤な有害事象に関する報告書」**を用いる。
3. 他施設が研究代表施設の場合、対応は研究代表施設の手順に従う。

**研究機関の長、研究責任者（研究代表者）への報告要否と報告期限**

|  | **軽症/中等症/重症（Grade1/2/3）** | | | **最重症（Grade4）** | | **死亡** | | **その他**  **医学的に**  **重要な**  **状態** |
| --- | --- | --- | --- | --- | --- | --- | --- | --- |
|  | **予測できる**  **（既知）** | **予測できない**  **（未知）** | | **予測できる**  **（既知）** | **予測できない**  **（未知）** | **予測できる**  **（既知）** | **予測できない**  **（未知）** |  |
|  | **入院**  **なし／あり** | **入院**  **なし** | **入院**  **あり** |  |  |  |  |  |
| **因果関係あり** | 報告  不要 | 報告  不要 | 初回報告  ：10日以内  追加報告  ：随時 | 一次報告：72時間以内  二次報告：7日以内  追加報告：随時 | | | |  |
| **因果関係なし** | 報告  不要 | 報告  不要 | 初回報告  ：10日以内^*^  追加報告  ：随時^*^ | 一次報告：72時間以内^*^  二次報告：7日以内^*^  追加報告：随時^*^ | | | |  |

^*^治療中または最終プロトコル治療日から30日以内のみ

# 共同研究機関への報告

1. 多施設共同研究の場合、研究責任者（研究代表者）は、有害事象／不具合が発現した研究機関の研究責任者、共同研究機関の研究責任者に以下を報告し、研究機関の長、倫理委員会への報告を依頼する。

| ①重篤な有害事象に関する報告書 |
| --- |

1. 研究機関の研究責任者は、研究機関の長の指示を受け、必要な措置を講じる。

# 侵襲を伴う研究の場合、研究によって生じた健康被害に対する補償の有無、内容

研究の実施に起因して研究対象者に健康被害が発生した場合、研究機関および研究責任者は治療その他必要な措置を講じる。健康被害に対する治療に係る医療費は、研究対象者の健康保険を適用し、金銭的な補償はない。

# 研究の実施に伴い、研究対象者の健康、子孫に受け継がれ得る遺伝的特徴等、重要な知見が得られる可能性がある場合、研究対象者に係る研究結果（偶発的所見を含む）の取扱い

本研究ではMRIの撮影および認知心理検査に伴い、偶発的な所見が得られる可能性がある。

MRI：脳腫瘍、脳膿瘍、脳出血、脳梗塞、脳血管動脈瘤、脳動静脈奇形などの緊急性を要する異常所見が発見される可能性がある。

認知心理検査：軽度認知機能障害など、認知機能低下を疑う結果が得られることがある。

検査結果の全員への回付は行わない。研究に参加する段階で明らかな異常所見が認められた場合の所見の回付希望の有無を調査し、文書に記録する。これら異常所見が認められた場合は、参加者の事前の希望に応じて、書面にて医療機関への受診を勧める。

# 試料・情報が同意を受ける時点では特定されない将来の研究のために用いられる可能性／他の研究機関に提供する可能性がある場合、その旨と同意を受ける時点において想定される内容

本研究で得られたデータ（MRI画像、認知機能検査、年齢・身長など被験者記録）は、倫理委員会で承認された研究において使用することが想定される。結果の転用の可否について、研究参加時に意思を確認し、これに従う。転用する研究が倫理委員会にすでに承認されたものでない場合、倫理委員会に再度申請する。

# 研究に関する情報公開の方法

# 研究の概要及び結果の登録

研究責任者は、公開データベース（UMIN）に研究概要を登録し、研究計画書変更、研究進捗に応じて適宜更新する。

ただし、研究対象者等の人権、研究者等の関係者の人権、知的財産保護のため非公開とする事項、個人情報保護の観点から研究に著しく支障が生じるため倫理委員会の意見を受け研究機関の長が許可した事項は非公開とする。

# 研究結果の公表

研究責任者は、研究終了後、研究対象者の個人情報保護に措置を講じた上で、遅滞なく研究結果を医学雑誌等に公表する。

結果の最終公表を行った場合、遅滞なく研究機関の長に報告する。

# 試料・情報等の保存・廃棄の方法

# 保存

| **保存する試料・情報等** | **保存期間** |
| --- | --- |
| ○研究に用いられる試料（検体） | 研究終了後廃棄 |
| ○研究に用いられる研究対象者情報（診療情報、検査データ、症例報告書等）  ○試料・情報の提供に関する記録、対応表  ○研究記録、手順書等 | 研究終了日から5年／結果公表日から3年  （いずれか遅い日） |

# 廃棄

研究責任者は、人体から取得した試料・情報等を廃棄する場合、匿名化する。

# 研究機関の長への報告内容、方法

研究責任者は、以下を研究機関の長に所定の様式により報告する。

・倫理的妥当性・科学的合理性を損なう事実に関する報告

・研究の実施の適正性若しくは研究結果の信頼を損なう事実若しくは情報又は損なうおそれのある情報を得た場合の報告

・研究の進捗状況及び有害事象発生状況の報告

・人体から取得された試料及び情報等の管理状況に関する報告

・研究終了及び研究結果概要の報告

# 研究計画書の変更

研究計画書を変更する場合、研究責任者は、倫理委員会の審査を経て研究機関の長の承認を得る。

研究計画書内容の変更を、改正・改訂の2種類に分けて取扱う。その他、研究計画書の変更に該当しない補足説明の追加をメモランダムとして区別する。

1. **改正（Amendment）**

研究対象者の危険を増大させる可能性のある、または主要評価項目に影響を及ぼす研究計画書の変更。各研究機関の承認を要する。以下の場合が該当する。

①研究対象者に対する負担を増大させる変更（採血、検査等の侵襲の増加）

②重篤な副作用の発現による除外基準等の変更

③有効性・安全性の評価方法の変更

④症例数の変更

1. **改訂（Revision）**

研究対象者の危険を増大させる可能性がなく、かつ主要評価項目に影響を及ぼさない研究計画書の変更。各研究機関の承認を要する。以下の場合が該当する。

①研究対象者に対する負担を増大させない変更（検査時期の変更）

②研究期間の変更

③研究者の変更

1. **メモランダム／覚え書き（Memorandum）**

研究計画書内容の変更ではなく、文面の解釈上のバラツキを減らす、特に注意を喚起する等の目的で、研究責任者から研究関係者に配布する研究計画書の補足説明。

# 研究の実施体制

# 研究機関の名称、研究責任者の氏名

研究責任者：

東北大学加齢医学研究所、臨床加齢医学研究分野、瀧靖之、教授、連絡先022-717-8559

# 研究分担者等の氏名・役割

(1)研究分担者：中瀬泰然、東北大学加齢医学研究所　臨床加齢医学分野、講師、022-717-8559

(2)研究協力者：真壁さやか、千葉真以子、阿部愛実、松平泉

東北大学スマート・エイジング学際重点研究センター、022-717-8559

小林恒輝、壹岐ひかり、朝岡陽香、舘脇康子

臨床加齢医学研究分野、022-717-8559

(3)試験薬管理者／試験機器管理者：中瀬泰然、臨床加齢医学分野、022-717-8559

(4)試料・情報等の保存・管理責任者：中瀬泰然、臨床加齢医学分野、連絡先022-717-8559

# 研究事務局、統計解析

(1)研究事務局：中瀬泰然　（講師）臨床加齢医学研究分野、022-717-8559

(2)統計解析責任者：中瀬泰然、臨床加齢医学分野、022-717-8559

(3)データ管理者：中瀬泰然、臨床加齢医学分野、022-717-8559

(4)データマネジメント従事者： 武藤達士、臨床加齢医学研究分野、022-717-8559

# 共同研究機関

1. 共同研究機関：あり（機関名を特定できる）

○機関名を特定できる場合

・機関名：大木製薬株式会社

・研究責任者等の氏名：川本宏和

# 研究に関する問合せ窓口

(1)研究対象者（参加者）の登録方法：中瀬泰然、加齢研機能画像医学分野、022-717-8559

(2)有害事象発生時の対応方法：中瀬泰然、加齢研機能画像医学分野、022-717-8559

# 引用文献

1. Mix JA, Crews Jr WD. An examination of the efficacy of Gingko biloba extract EGb761 on the neuropsychologic functioning of cognitively intact older adults. J Altern Comp Med 2000;6:219-229.
2. Dodge HH, et al. A randomized placebo-controlled trial of Gingko biloba for the prevention of cognitive decline. Neurology 2008;70(19):1809-1817.
3. Moulton PL, et al. The effect of Gingko biloba on memory in healthy male volunteers. Physiol Behav 2001;73:659-665.
4. Chen F, et al. Systemic and cerebral exposure to and pharmacokinetics of flavonols and terpenelactones after dosing standardized Gingko biloba leaf extracts to rats via different routes of administration. Br J Pharmacol 2013;170:440-457.
5. Matsumura S, et al. Inhibitory activities of sesame seed extract and its constituents against β-secretase. Nat Product Com 2016;11(11):1671-1674.
6. Matsumura S, et al. Inhibitory activities of essential oil obtained from turmeric and its constituents against β-secretase. Nat Product Com 2016;11(12):1785-1788.
7. Iwamoto K, et al. Using turmeric oil as a solvent improves the distribution of sesamin-sesamolin in the serum and brain of mice. Lipids 2019;54(5):311-320.
8. Iwamoto K, et al. Mixing Ginkgo biloba extract with sesame extract and turmeric oil increases bioavailability of ginkgolide A in mice brain. J Oleo Sci 2019;68(9):923-930.
9. 認知症疾患診療ガイドライン2017
10. Zhang HF, et al. An overview of systematic reviews of Gingko biloba extracts for mild cognitive impairment and dementia. Front Aging Neurosci 2016;8:276.

# Appendix

・説明・同意文書
