# Supplementary material for: Efficacy of a mixture of Ginkgo biloba, sesame, and turmeric on cognitive function in healthy adults: Study protocol for a randomized, double-blind, placebo-controlled trial
Source: PLoS One. 2023 Mar 15;18(3):e0280549. doi: 10.1371/journal.pone.0280549 (PMC10016651; doi:10.1371/journal.pone.0280549)
Supplement: Attachment — Submitted filename: response.docx [file pone.0280549.s004.docx]

Response to editor and reviewers.

> I agree with reviewer 2 that the inclusion and exclusion criteria can be presented as text, rather than as bullet points, and that the quality figure 1 needs improvement. I also agree that further details on blinding and randomization could be provided.

Thank you very much for your effective comments. We rewrote the inclusion and exclusion criteria in text style (p5 line19 – line29). Figure 2, previous Fig 1, was corrected the quality. We described the section of Randomization more precisely (p7 line8 – line13).

> However, I will leave it up to you whether to make changes to your title and keywords, and also whether you think it is appropriate to include any discussion of CAM treatments for various diseases.

Thank you very much for your suggestion. We changed the title following comment of Reviewer #2 (p1 line1 – line2). However, because our study is not for medical treatment, we did not include keywords, “integrative medicine” and “phytotherapy”. At the begging of the introduction section, we briefly mentioned the meaning of CAM following Reviewer #2’s comment (p3 line2 – line5).

>2. We note that the grant information you provided in the ‘Funding Information’ and ‘Financial Disclosure’ sections do not match.

We corrected and matched the funding information and financial disclosure (p1 line13 – line23).

>3. Thank you for providing the following Funding Statement: “I have read the journal's policy and the authors of this manuscript have the following competing interests: HK is employee of Ohki Pharmaceutical Company.”

We note that one or more of the authors is affiliated with the funding organization, indicating the funder may have had some role in the design, data collection, analysis or preparation of your manuscript for publication; in other words, the funder played an indirect role through the participation of the co-authors.

If the funding organization did not play a role in the study design, data collection and analysis, decision to publish, or preparation of the manuscript and only provided financial support in the form of authors' salaries and/or research materials, please review your statements relating to the author contributions, and ensure you have specifically and accurately indicated the role(s) that these authors had in your study in the Author Contributions section of the online submission form. Please make any necessary amendments directly within this section of the online submission form.  Please also update your Funding Statement to include the following statement: “The funder provided support in the form of salaries for authors [insert relevant initials], but did not have any additional role in the study design, data collection and analysis, decision to publish, or preparation of the manuscript. The specific roles of these authors are articulated in the ‘author contributions’ section.”

If the funding organization did have an additional role, please state and explain that role within your Funding Statement.

Actually, HK and FT do not participate in execution of the study. As they will only provide study materials, we removed their name from authors list and put their name in the acknowledge section. They, of course, agreed our decision. We rewrote the COI statement (p1 line4 – line5, p11 line15 – line17). Additionally, one listed author, YZ, has not been contributing to this study anymore. So that we deleted this name from authors list.

>4. Your ethics statement should only appear in the Methods section of your manuscript. If your ethics statement is written in any section besides the Methods, please delete it from any other section.

Following your comment, we deleted the ethics statement from abstract (p2 line10). We included the ethics statement only in the methods section (p9 line25 – p10 line11).

5. Please review your reference list to ensure that it is complete and correct. If you have cited papers that have been retracted, please include the rationale for doing so in the manuscript text, or remove these references and replace them with relevant current references. Any changes to the reference list should be mentioned in the rebuttal letter that accompanies your revised manuscript. If you need to cite a retracted article, indicate the article’s retracted status in the References list and also include a citation and full reference for the retraction notice.

Following Reviewer #2’s comment, we wrote the status of CAM and cited additional references which support our discussion (p3 line2 – line5).

>SPIRIT schedule is missing:

We included SPIRIT figure in the manuscript as Figure 1. (p5 line8 – line14)

>Completed SPIRIT checklist is missing:

We completed SPIRIT checklist and uploaded. Moreover, following SPIRIT lists, we newly described additional descriptions in the text (p5 line13, p6 line16, p7 line8 – line13, p7 line22 – line23, p8 line24 – line29, p10 line5 – line11).

Throughout text, we changed some words and sentences for keeping consistency of the manuscript. All changed parts were highlighted in red.

To Reviewer #1,

Thank you very much. We appreciate to your encouraging comments.

Reviewer #2:

1. It is suggested to change your title from “Efficacy of a mixture of Ginkgo biloba extract, sesame extract, and turmeric oil on cognitive function in healthy adults: protocol for a 24-week, randomized, double-blind, placebo-controlled trial” to “Efficacy of a mixture of Ginkgo biloba, sesame, and turmeric on cognitive function in healthy adults: study protocol for a randomized, double-blind, placebo-controlled triall.

Thank you very much for your effective suggestion. Now, we changed the words of title following your comment (p1 line1 – line2).

2. “Integrative medicine” and “phytotherapy” should be added to the keywords. “Ginkgo biloba extract; sesame extract; turmeric oil” should be changed to “Ginkgo biloba; sesame; turmeric”.

Thank you for your comments. We corrected the material names in the keywords following your comment (p2 line30 – line31). However, because our study is focusing not on the medical effect of GBE but on the influence of GBE on the cognitive function of healthy adults, we did not include these two keywords you suggested.

3. At the beginning of the introduction, a paragraph on CAM position for treatment of various diseases and the necessity for an evidence-based approach in CAM should be added. The below-mentioned references are strongly recommended to be cited there:
- Paudyal, Vibhu, et al. "Complementary and alternative medicines use in COVID-19: A global perspective on practice, policy and research." Research in Social and Administrative Pharmacy 18.3 (2022): 2524-2528.
- Hashemi, Monire Seyed, et al. "Efficacy of pomegranate seed powder on glucose and lipid metabolism in patients with type 2 diabetes: a prospective randomized double-blind placebo-controlled clinical trial." Complementary Medicine Research 28.3 (2021): 226-233.
- Wu, Jinhui, et al. "Complementary and Alternative Medicine Use by ADHD Patients: A Systematic Review." Journal of Attention Disorders (2022): 10870547221111557.

Thank you for your suggestion. We mentioned the current situation of CAM at the beginning of introduction section including references as you suggested (p3 line2 – line5).

4. The inclusion and exclusion criteria should be re-written as text (not bullet points).

Thank you for your suggestion. We rewrote the inclusion and exclusion criteria in text style (p5 line19 – line29).

5. In Table 1, “Piper longum extract” has been written instead of “turmeric oil”. What about its discrepancies? In addition, it is suggested to use common name or scientific name, through the manuscript.

Thank you for your careful reading. That words were our mistake. We corrected the name of the material (p6 line4 – 5 in Table 1).

6. Blinding should be described in detail. Also, details of randomization should be expanded.

Thank you for your comment. We described the blinding procedure and the method of randomization more precisely (p7 line8 – line13).

7. Figure 1 has not acceptable quality.

We rewrote Figure 2 (former Figure 1), more carefully.
